# Cyclooxygenase-1 mediates neuroinflammation and neurotoxicity in a mouse model of retinitis pigmentosa

**DOI:** 10.1186/s12974-020-01993-0

**Published:** 2020-10-15

**Authors:** Wei Yang, Guoyin Xiong, Bin Lin

**Affiliations:** 1 School of Optometry, The Hong Kong Polytechnic University, Hung Hom, Kowloon, Hong Kong; 2 Department of Ophthalmology, University of Hong Kong, Pokfulam, Hong Kong

**Keywords:** rd10 mice, Photoreceptors, Neuroinflammation, COX-1, EP2 receptor

## Abstract

**Background:**

Retinitis pigmentosa (RP) is a group of inherited eye disorders with progressive degeneration of photoreceptors in the retina, ultimately leading to partial or complete blindness. The mechanisms underlying photoreceptor degeneration are not yet completely understood. Neuroinflammation is reported to play a pathological role in RP. However, the mechanisms that trigger neuroinflammation remain largely unknown. To address this question, we investigated the role of cyclooxygenase-1 (COX-1), a key enzyme in the conversion of arachidonic acid to proinflammatory prostaglandins, in the rd10 mouse model of RP.

**Methods:**

We backcrossed COX-1 knockout mice (COX-1^−/−^) onto the rd10 mouse model of RP and investigated the impact of COX-1 deletion on neuroinflammation in the resulting COX-1^−/−^/rd10 mouse line, using a combination of immunocytochemistry, flow cytometry, qPCR, ELISA, and a series of simple visual tests.

**Results:**

We found that genetic ablation or pharmacological inhibition of COX-1 alleviated neuroinflammation and subsequently preserved retinal photoreceptor and function and visual performance in rd10 mice. Moreover, we observed that the pharmacological inhibition of the prostaglandin E2 (PGE2) EP2 receptors largely replicated the beneficial effects of COX-1 deletion, suggesting that EP2 receptor was a critical downstream effector of COX-1-mediated neurotoxicity in rd10 mice.

**Conclusion:**

Our data suggest that the COX-1/PGE2/EP2 signaling pathway was partly responsible for significantly increased neuroinflammation and disease progression in rd10 mice, and that EP2 receptor could be targeted therapeutically to block the pathological activity of COX-1 without inducing any potential side effects in treating RP patients.

**Supplementary information:**

The online version contains supplementary material available at 10.1186/s12974-020-01993-0.

## Background

Retinitis pigmentosa (RP) is a heterogeneous group of inherited disorders caused by a number of gene mutations [1, 2]. More than 3000 mutations in over 60 genes have so far been causally associated with RP [3]. Genetic defects in a majority of RP genes result in initial death of rod photoreceptors followed by cone photoreceptors [1, 2]. Progressive degeneration of photoreceptors ultimately leads to partial or complete blindness that affects 1 in 4000 humans worldwide [1]. However, the molecular mechanisms by which these mutations lead to photoreceptor death are still not fully understood. No effective treatment is currently available for RP patients.

Neuroinflammation has been reported to be involved in RP progression [4, 5]. We and others have previously demonstrated that microglia-mediated inflammation is a driving force that promotes neurodegeneration in RP animal models [6–9]. However, the mechanisms underlying the chronic neuroinflammation remain largely unknown in RP. Two different isoforms of cyclooxygenase (COX), COX-1 and COX-2, catalyze the conversion of arachidonic acid to prostaglandins, which are involved in physiological and pathological processes. Prostaglandin E2 (PGE2), one of the prostaglandins downstream of COX, plays a key role in the generation of inflammatory responses [10–12]. COX-1, which has its predominant localization in microglia, has emerged as a prominent player in neuroinflammation and neurodegeneration in the brain [13]. COX-1 is reported to facilitate pro-inflammatory prostaglandin upregulation in several models of neurodegenerative disorders [13–15]. Conversely, COX-1 inhibition is found to be beneficial in Alzheimer’s disease [16]. These previous findings consistently support an important role for COX-1 in neuroinflammation in brain disorders. However, it remained largely unknown whether COX-1 and its downstream prostaglandin signaling underlay neuroinflammation in RP.

To address the potential role of COX-1 in RP progression, we backcrossed COX-1 knockout mice (COX-1^−/−^) onto rd10 mice, a well-characterized mouse model of RP [17, 18], and investigated the impact of COX-1 deletion on neuroinflammation in the resulting COX-1^−/−^/rd10 mouse line. Alternatively, we pharmacologically inhibited COX-1 with the COX-1 specific inhibitor SC-560 in rd10 mice. We found that either COX-1 deletion or inhibition consistently reduced inflammatory responses and the production of inflammatory mediators, which subsequently resulted in the preservation of retinal photoreceptor and function and visual performance in rd10 mice. These observations confirmed the involvement of COX-1 in mediating neuroinflammation in RP.

PGE2 elicits inflammatory processes through binding to downstream membrane-specific G-protein coupled EP1-4 receptors [19]. Among them, EP2 receptor is highly expressed in macrophages and is also a crucial mediator of brain inflammation in the setting of neurodegeneration [10–12]. However, it remained unclear whether EP2 receptor participated in COX-1-mediated neurotoxicity in the rd10 retina. To evaluate its role, we treated rd10 mice with TG6-10-1, a potent antagonist of EP2 receptor [10]. We found that systemic administration of TG6-10-1 largely counteracted the detrimental effects of COX-1 and mitigated disease progression, indicating that PGE2 signaling through EP2 receptor was probably involved in regulating proinflammatory responses in rd10 mice. Collectively, we demonstrated that the COX-1/PGE2/EP2 signaling pathway played an important role in mediating neuroinflammation and disease progression in rd10 mice. Our findings suggested that selective inhibition of EP2 signaling pharmacologically might be potential therapeutic targets to block the pathological activity of COX-1 without compromising its beneficial effects in RP.

## Methods

### Animals

C57BL/6J mice (Stock no: 000664), transgenic CX3CR1^GFP/GFP^ mice (Stock no: 005582), and rd10 mice (Stock no: 004297) were obtained from Jackson Laboratory (Bar Harbor, ME, USA). Homozygous knockout of COX-1 (COX-1^−/−^) mice were kindly provided by Dr. George Tipoe at University of Hong Kong [20]. Rd10 mice were backcrossed with COX-1^−/−^ mice, and the littermates of both sexes from rd10/COX-1^−/−^ mice were used for experiments. Rd10/CX3CR1^+/GFP^ mice were previously generated by backcrossing rd10 mice with CX3CR1^GFP/GFP^ mice, in which microglia are fluorescently labeled after replacing the Cx3cr1 gene with the gene encoding enhanced green fluorescent protein (EGFP) [6]. The genotypes of mouse litters were determined by PCR and confirmed by Southern blot analysis of genomic DNA from tail biopsies. Animals were bred and maintained at the Centralised Animal Facilities (CAF) of The Hong Kong Polytechnic University on a 12-h light-dark cycle with a room illumination of around 50 lx and water and food ad libitum. All experimental procedures were approved by the Animal Subjects Ethics Sub-committee (ASESC) of Hong Kong Polytechnic University and conducted in accordance with the Association for Research in Vision and Ophthalmology (ARVO) statement for the use of animals.

TG6-10-1 (5 mg/kg; Cayman Chemical, Ann Arbor, USA), a potent antagonist of EP2 receptor, was administered via intraperitoneal injection to rd10/CX3CR1^+/GFP^ or rd10 mice twice a day (12 h apart), starting from P16. TG6-10-1 was dissolved in a formulation solution consisting of 10% DMSO, 40% water, and 50% polyethylene glycol. Control groups received the same volume of the formulation solution at the same time points. SC-560 (10 mg/kg in 25% DMSO, Sigma), a specific inhibitor of COX-1, was administered intraperitoneally to rd10 or rd10/CX3CR1^+/GFP^ mice daily, starting from P16. Rd10 or rd10/CX3CR1^+/GFP^ mice in control groups received the same volume of 25% DMSO at the same time points. Butaprost (Cayman chemical company, USA), an EP2 agonist, was dissolved in a formulation solution consisting of 10% DMSO, 40% water, and 50% polyethylene glycol, and was administered via intraperitoneal injection to COX-1^−/−^/rd10 mice daily (4 mg/kg/per day). Control groups received the same volume of the formulation solution at the same time points.

### Immunocytochemistry and confocal imaging

Animals were sacrificed with an overdose of sodium pentobarbital. Eyes were quickly enucleated after a reference point was made to label the superior pole and the retinas were dissected free of vitreous and sclera in carboxygenated Ames’ medium (Sigma-Aldrich, St. Louis, USA), and then fixed in 4% paraformaldehyde (PFA) in 0.1 M phosphate buffer (PB), pH 7.4 for 0.5–1 h. Some retinas were sectioned serially at a thickness of 10–12 μm using a cryostat. Some retinal sections were cut and stained with hematoxylin and eosin (H&E). Both whole-mounted retinas and cross sections were blocked in a solution containing 3% normal goat serum (NGS), 1% bovine serum albumin (BSA), and 0.3% Triton X-100 in PBS (pH 7.4) for 1 h. Primary antibodies used were rabbit anti-red/green opsin (1:500, Chemicon, Temecula, USA), rabbit anti-Iba-1 (1:500, Wako, Japan), and rabbit anti-COX-1 antibody (1:100; Abcam, USA).

The primary antibodies were diluted with a blocking solution (1% NGS, 1% BSA, 0.1% Triton X-100 in PBS) and applied to sections or whole-mounted retinas from overnight to 3 days at 4 °C. After blocking and rinsing, a secondary antibody conjugated to either Alexa 488 (1:500; Invitrogen, USA) or Alexa 594 (1:500; Invitrogen, USA) was applied to sections or whole-mounted retinas for 2 h at room temperature. Sections and whole-mounted retinas were rinsed, and cover slipped in Vectashield mounting medium (Vector Laboratories, Burlingame, USA).

Confocal micrographs of fluorescent specimens from flat-mounted retinas and retinal sections were captured using a Zeiss LSM 800 confocal microscope with Airyscan (Carl Zeiss, Germany). Plan-Apochromat 63×/1.4 or 40×/1.4 oil immersion objective lenses were used. Images scale was calibrated, and if necessary, brightness and contrast were adjusted using Photoshop CS6 software (Adobe Systems, USA).

### Electroretinographic analysis

Electroretinography (ERGs) were recorded using an Espion ERG Diagnosys machine (Diagnosys, Littleton, MA) as previously described by us [21]. Briefly, flash ERG was measured using a gold wire corneal electrode, a forehead reference electrode, and a ground electrode near the tail. Scotopic, rod-mediated responses were obtained from dark-adapted animals at 3 cd-s/m^2^. Photopic, cone-mediated responses were performed following 10-min light adaptation on the background light intensity of 30 cd/m^2^. Recordings were obtained at the light intensity of 3 cd-s/m^2^. Fifteen waveforms from each animal were recorded and the values were averaged. The ERG a-wave amplitudes were measured from the baseline to the negative peak and the b-wave was measured from the trough of the a-wave to the peak of the first positive wave or, when the a-wave was not present, from baseline to the peak of the first positive wave.

### Optokinetic tracking

Optokinetic tracking was performed using a virtual optokinetic system (OptoMotry, CerebralMechanics, Canada) as previously described by us [6, 22]. Mice were placed on a platform positioned in the middle of an arena created by a quad-square of computer monitors. Vertical sine wave gratings (100% contrast) were projected on the computer monitors. The spatial frequencies tested were 0.05, 0.075, 0.1, 0.2, 0.3, 0.4, 0.5, 0.6 cycle per degree (cpd), at a constant speed of 12 degree per second. The image of eye movements was monitored by an infrared-sensitive small camera.

### Flow cytometry and real-time qPCR

Flow cytometry and cell sorting was performed as previously described by us [6]. In brief, two retinas from each mouse were pooled together, minced and incubated in 2 ml of HBSS containing 0.5 mg/ml papain (Worthington, Lakewood, NJ, USA) for 20 min at 37 °C. The reaction was then quenched using 2 ml of minimal essential medium containing 5% horse serum and 200 U/ml DNAseI (Sigma-Aldrich, USA). The dissociated retinal cells were incubated with fluorochrome-conjugated antibody to CD11b (FITC anti-CD11b, 1:250; BD Biosciences, San Jose, CA, USA) in FACS buffer on ice for 20 min. FITC-CD11b-labeled microglial cell populations were separated using a Becton-Dickinson FACSAria III Cell Sorter. For the sorting of microglial cell populations, we followed a standard protocol as previously described by others [23]. Retinal samples were gated positive for FITC-CD11b-positive microglial cells, after exclusion of debris and doublets. We also followed the same steps to sort GFP-expressing microglial cells from Rd10/CX3CR1^+/GFP^ mice or CX3CR1^+/GFP^ mice without incubation with FITC anti-CD11b. Total RNA from FACS purified microglia was reverse transcribed and amplified with TagMan pre-designed real-time PCR assays (Applied Biosystems). Quantitative PCR was performed on a MyIQ thermal cycler (Biorad) with TagMan Master Mixes (Applied Biosystems). Each sample was run in quadruplicate in each assay. Beta-actin was used as the endogenous control.

### Enzyme-linked immunosorbent assay measurement

Enzyme-linked immunosorbent assay (ELISA) for PGE2 (Enzo Life Sciences, USA), Cox-1 (1:500, Cayman Chemicals, USA), TNF-α (PeproTech, Rocky Hill, NJ), and IL-1β (PeproTech, Rocky Hill, NJ) expression in retinas were performed using ELISA kits, respectively, according to manufacturer’s instructions as previously described by us [21, 22]. The specificity of all the antibodies used for ELISA in mouse has been validated by the manufacturers.

### BV-2 cell culture

BV-2 cells, a murine microglial cell line, were kindly provided by Prof Xu Haiwei at Southwest Eye Hospital, Chongqing, China [24]. In brief, BV2 cells were seeded into six-well plates at a concentration of 1 × 10^5^ cells/well in Dulbecco’s modified Eagle’s media (DMEM) containing 10% fetal bovine serum, 100 U/ml penicillin, and 100 μg/ml streptomycin (Gibco) in a humidified incubator containing 5% CO_2_, and 95% air, as previously described by us and others [24, 25]. The culture medium was changed to fresh medium for routine culture before the cells were exposed to lipopolysaccharide (LPS) exposure (1 μg/ml). SC560 (3 μM) was added to media 30 min ahead of LPS treatment. After 24-h incubation, culture media were collected for PGE2 ELISA assay, and some cells were collected for RNA extraction and subsequent rt-qPCR (LightCycler® 480 SYBR Green I Master).

### Data analysis

For measurement of the ONL thickness, vertical sections passing through the optic nerve head were used for counting the number of photoreceptor nuclear rows stained with 4′,6-diamidino-2-phenylindole dihydrochloride (DAPI). Measurements were taken at 1 mm from the optic nerve on both sides. To measure the length of cone outer and inner segments, we chose the sections that passed through the optic nerve head and stained the sections with red/green opsins. Three sections per retina were examined. Quantification of surviving cones was conducted in retinal whole-mounts stained with red/green opsins. Sampling areas were two 240 μm × 240 μm squares along the dorsal-ventral axis of retinal whole-mounts, 1 mm from the optic nerve on both sides. The raw counts were then converted into cells/millimeter^2^.

### Statistical analyses

All statistical analyses were performed in GraphPad Prism 7.0 (GraphPad Software). Statistical analyses were performed using ANOVA followed by Bonferroni’s and Dunnett’s post hoc tests for pairwise comparisons. A *p* value < 0.05 was considered statistically significant. All data are displayed as the mean ± SD.

## Results

### COX-1 is upregulated in the rd10 retina

**Fig. 1:**
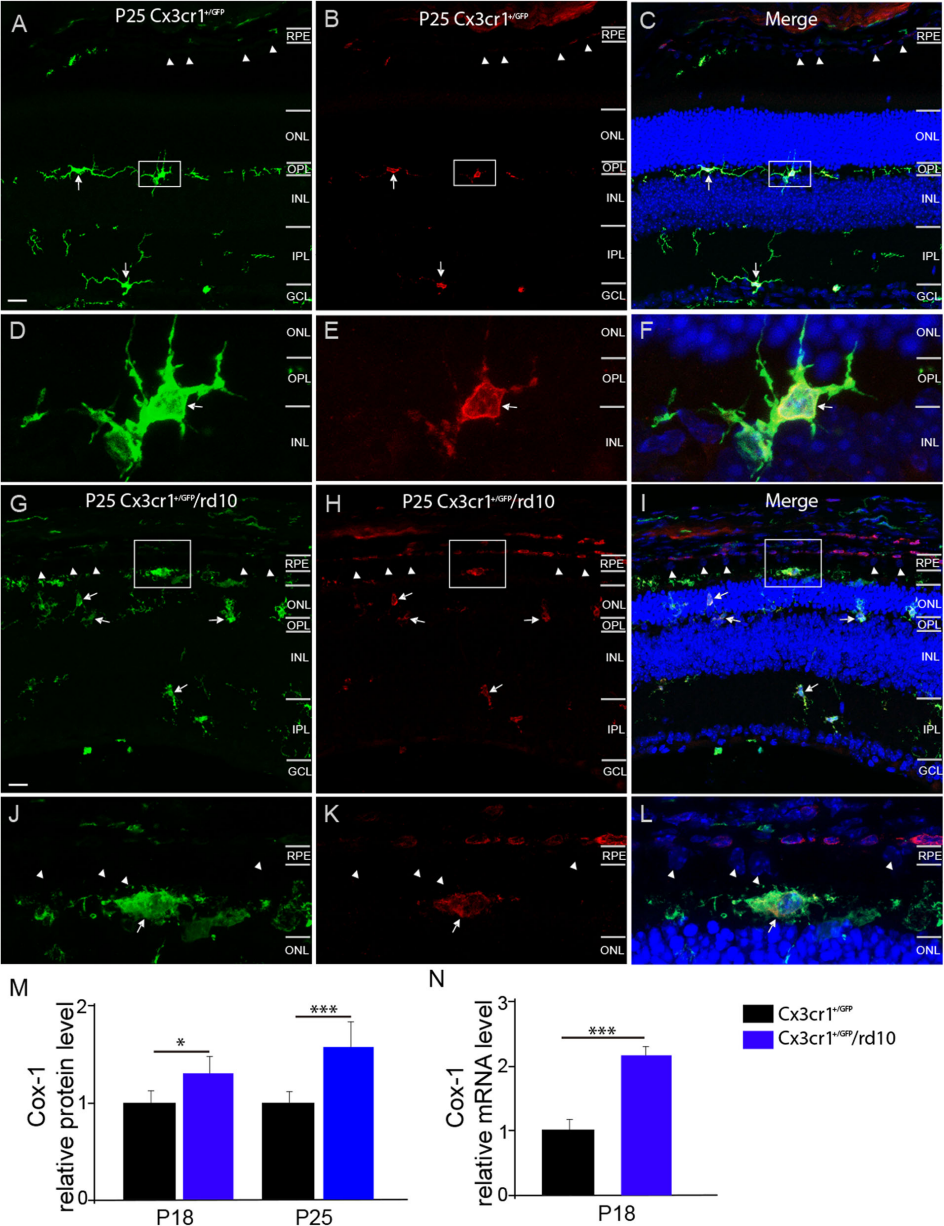
The upregulation of COX-1 expression in the rd10 retina. **a**–**f** Retinal sections from the dorsal retina at about 1 mm away from the optic nerve head along the dorsal-ventral axis of P25 CX3CR1^+/GFP^ (**a–c**) and CX3CR1^+/GFP^/rd10 (**g**–**i**) mice were stained with an antibody specific for COX-1 (red). **d**–**f** Highly magnified images from the boxed regions in **a**, **b,** and **c**. **j**–**l** Highly magnified images from the boxed regions in **g**, **h,** and **i**. Arrows indicate colocalization between GFP-expressing microglia (green) and COX-1 signals (red), and arrowheads point to retinal pigment epithelial cells. Cell nuclei were stained with 4′,6-diamidino-2-phenylindole dihydrochloride (DAPI) (blue). *RPE* retinal pigment epithelium, *ONL* outer nuclear layer, *OPL* outer plexiform layer, *INL* inner nuclear layer, *IPL* inner plexiform layer, *GCL* ganglion cell layer. Scale bar, 20 μm in **a–**c and **g**–**i**. **m** ELISA quantification of COX-1 protein levels from P18 and P25 CX3CR1^+/GFP^ and CX3CR1^+/GFP^/rd10 mouse retinas (P18: WT versus rd10, *t*(8) = 2.31, *p* = 0.025; P25: WT versus rd10, *t*(8) = 4.92, *p* = 0.00058). **n** qPCR analyses of COX-1 mRNA levels in sorted primary microglial cells by fluorescence activated cell sorting (FACS) from P18 CX3CR1^+/GFP^ and CX3CR1^+/GFP^/rd10 mouse retinas (WT versus rd10, *t*(8) = 4.39, *p* = 0.00096). Each bar represents the mean value (± s.d.) calculated from 5 mice and normalized to the value for WT group, which was set to 1.00. *p < 0.05, ***p < 0.001 vs. age-matched WT control

To establish the involvement of COX-1 in modulating proinflammatory responses in RP, we investigated whether COX-1 was dysregulated in the retina of rd10 mice, a well-characterized mouse model of RP. Rd10 mice carry a missense mutation in the beta subunit of rod-specific phosphodiesterase gene 6 (PDE6β) in exon 13, which causes the massive degeneration of rod photoreceptors followed by gradual degeneration of cones. Microglia are localized in the inner half of the retina as well as the outer plexiform layer in WT mouse retinas (Fig. 1a). We previously demonstrated that activated retinal microglia migrate into the outer nuclear layer (ONL) from postnatal day 16 (P16) onward in the rd10 retina [6]. Therefore, we chose several time points right after P16 for analyzing COX-1 expression. COX-1 is predominantly expressed in microglia in the brain [13]. Therefore, we first identified whether microglia in the mouse retina expressed COX-1 by using an antibody specific for COX-1. Notably, we observed that GFP-expressing microglial cells co-localized with COX-1 isoform in CX3CR1^+/GFP^ WT (Fig. 1a–c, d–f, arrows) and CX3CR1^+/GFP^/rd10 (Fig. 1g–i, j–l, arrows) mouse retinas, indicating the expression of COX-1 in the microglia of mouse retinas. In addition, ELISA quantification showed that COX-1 was significantly upregulated in the CX3CR1^+/GFP^/rd10 retina compared to CX3CR1^+/GFP^ WT mouse retinas (Fig. 1m). To confirm whether COX-1 was upregulated in the microglia of rd10 retinas, we performed qPCR in sorted primary microglia from CX3CR1^+/GFP^/rd10 by using flow cytometry. Indeed, qPCR analysis confirmed upregulation of COX-1 transcripts in the microglia of CX3CR1^+/GFP^/rd10 mouse retinas relative to those in CX3CR1^+/GFP^ WT (Fig. 1n). Together, our data demonstrated upregulation of COX-1 in the rd10 retina, suggesting the possible involvement of COX-1 in regulating neuroinflammation in the rd10 retina.

### COX-1 deletion reduces microglia activation and alleviates neuroinflammation in rd10 mice

**Fig. 2:**
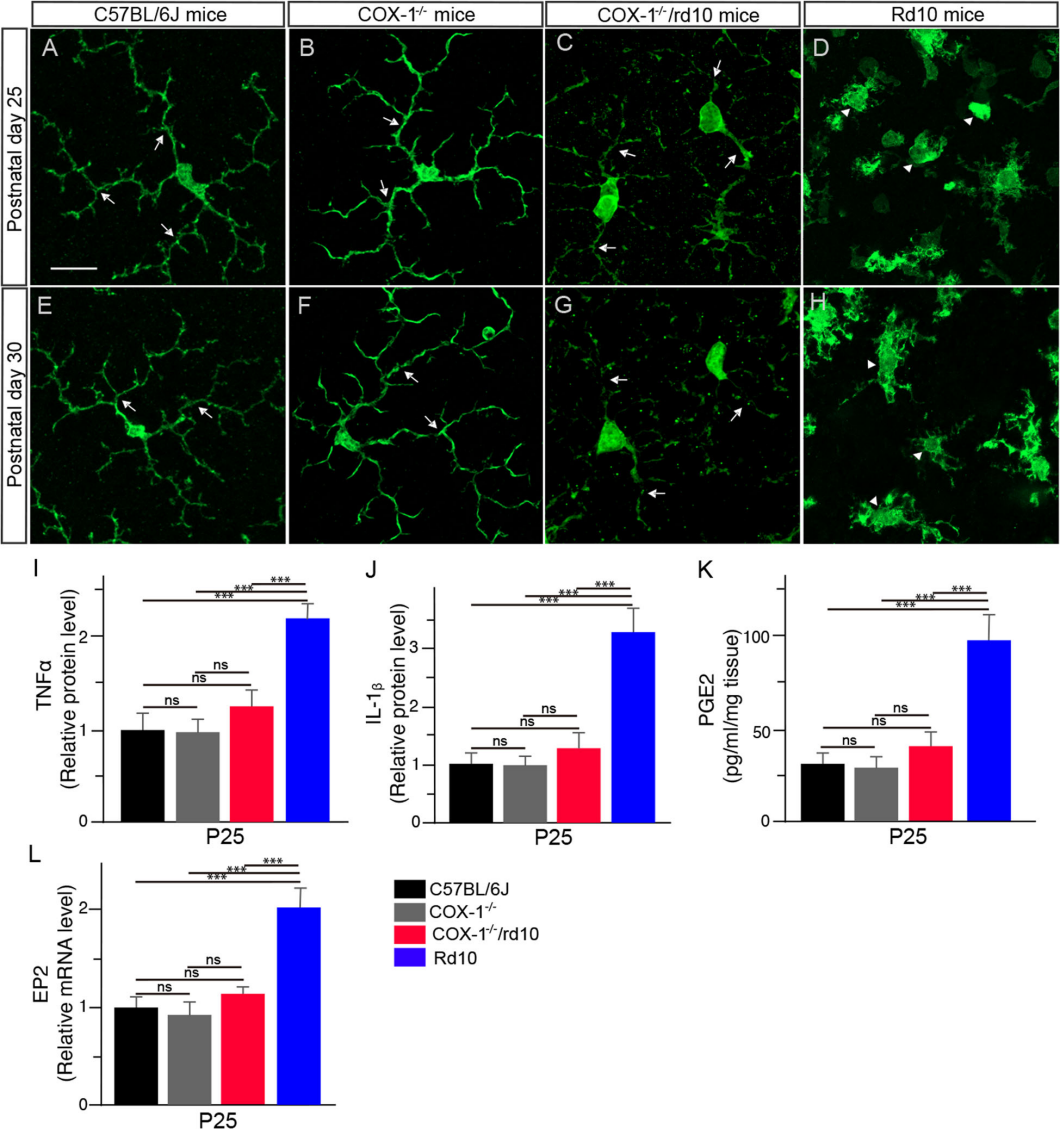
COX-1 deletion reduces microgliosis in rd10 mice. **a**–**h** Retina flat mounts were stained with an antibody against Iba-1, a marker for microglia. Confocal images from the dorsal retina at about 1 mm away from the optic nerve head along the dorsal-ventral axis show that microglia have a ramified morphology in P25 and P30 WT retinas (**a**, **e**, arrows). Similarly, microglia show a ramified morphology in P25 and P30 COX-1^−/−^ mouse retinas (**b**, **f**, arrows). Microglia display an amoeboid morphology with few processes and rounded cell bodies in P25 and P30 rd10 retinas (**d**, **h**, arrowheads), whereas microglia maintain a ramified appearance in age-matched COX-1^−/−^/rd10 mouse retinas (**c**, **g**, arrows). Scale bar: 20 μm. **i**–**l** ELISA analyses of TNF-α (**i**; *F*(3, 20) = 40.19, *p* = 1.2 × 10^−8^; WT versus COX-1^−/−^, *p* = 0.52; WT versus rd10, *p* = 1.3 × 10^−5^; WT versus COX-1^−/−^/rd10, *p* = 0.198; COX-1^−/−^ versus rd10, *p* = 1.1 × 10^−5^; COX-1^−/−^ versus COX-1^−/−^/rd10, *p* = 0.11; rd10 versus COX-1^−/−^/rd10, *p* = 1.2 × 10^−5^), IL-1β (**j**; *F*(3, 20) = 15.59, *p* = 1.8 × 10^−5^; WT versus COX-1^−/−^, *p* = 0.71; WT versus rd10, *p* = 3.5 × 10^−5^; WT versus COX-1^−/−^/rd10, *p* = 0.286; COX-1^−/−^ versus rd10, *p* = 1.3 × 10^−5^; COX-1^−/−^ versus COX-1^−/−^/rd10, *p* = 0.37; rd10 versus COX-1^−/−^/rd10, *p* = 0.0005), and PGE2 (**k**; *F*(3, 20) = 58.85, *p* = 4.2 × 10^−10^; WT versus COX-1^−/−^, *p* = 0.71; WT versus rd10, *p* = 2.4 × 10^−7^; WT versus COX-1^−/−^/rd10, *p* = 0.20; COX-1^−/−^ versus rd10, *p* = 4.8 × 10^−6^; COX-1^−/−^ versus COX-1^−/−^/rd10, *p* = 0.24; rd10 versus COX-1^−/−^/rd10, *p* = 4.8 × 10^−7^) protein expression levels in P25 WT, rd10, and COX-1^−/−^/rd10 mouse retinas. **l** qPCR analyses of EP2 mRNA levels in sorted primary microglial cells labeled with FITC-CD11b by FACS from P25 WT, rd10, and COX-1^−/−^/rd10 mouse retinas (*F*(3, 20) = 25.62, *p* = 4.7 × 10^−7^; WT versus COX-1^−/−^, *p* = 0.56; WT versus rd10, *p* = 1.4 × 10^−6^; WT versus COX-1^−/−^/rd10, *p* = 0.087; COX-1^−/−^ versus rd10, *p* = 1.2 × 10^−5^; COX-1^−/−^ versus COX-1^−/−^/rd10, *p* = 0.11; rd10 versus COX-1^−/−^/rd10, *p* = 0.00033). Results are presented as means ± SDs (*n* = 6 animals/each group). *ns* not significant and ***p < 0.001

To confirm the involvement of COX-1 in modulating inflammatory responses in the rd10 retina, we backcrossed COX-1 knockout mice (COX-1^−/−^ mice) with rd10 mice and assessed the effect of COX-1 deletion on microglia activation and neuroinflammation in the resulting COX-1^−/−^/rd10 mouse retina. Microglia display a ramified morphology in the WT (Fig. 2a, e, arrows) and COX-1^−/−^/ mouse retina (Fig. 2b, f, arrows), whereas microglia underwent gradual morphological transformation from a highly ramified morphology into an amoeboid shape with retracted processes in the rd10 retina (Fig. 2d, h, arrowheads). In the COX-1^−/−^/rd10 retina, however, we found that microglia maintained a ramified morphology (Fig. 2c, g, arrows), indicating the suppression of microglia activation after COX-1 deletion. After COX-1 deletion, microglial cells still display a hypertrophic morphology in the rd10 retina, indicating some degree of activation, which is probably contributed by other regulating mechanisms and signalling pathways are still involved in regulating neuroinflammation [26]. Additionally, we observed downregulation of inflammatory mediators including tumor necrosis factor-alpha (TNF-α) (Fig. 2i) and interleukin-1 beta (IL-1β) in the COX-1^−/−^/rd10 mouse retina (Fig. 2j), which confirmed a significant reduction in neuroinflammation. Moreover, we treated rd10 mice with SC-560, a specific inhibitor of COX-1, and examined whether COX-1 inhibition could recapitulate the beneficial effects observed in the COX-1^−/−^/rd10 mice. Interestingly, we found that pharmacological inhibition of COX-1 attenuated microglia activation and reduced the levels of proinflammatory mediators (Supplementary Figure 1). Taken together, the results from COX-1 deletion and pharmacological inhibition consistently showed suppression of microglia activation and alleviation of neuroinflammation in rd10 mice.

Moreover, we observed that prostaglandin E2 (PGE2) was significantly upregulated in the rd10 retina (Fig. 2k). However, COX-1 deletion led to a significant reduction in disease-elevated PGE2 in COX-1^−/−^/rd10 retinas relative to rd10 control retinas (Fig. 2k). These results indicated that COX-1 deletion abrogated PGE2 elevation in the rd10 retina, suggesting that COX-1 primarily droved the observed increase in PGE2 in the rd10 retina. The receptor EP2, one of PGE2 downstream G-protein coupled receptors, is highly expressed in microglia in the brain [10–12]. To examine whether downregulation of PGE2 affected EP2 expression in the microglia of the COX-1^−/−^/rd10 retina, we performed qPCR in sorted primary microglial cells by using flow cytometry. We found that EP2 transcripts were significantly downregulated in microglia from COX-1^−/−^/rd10 mouse retinas compared to those from rd10 control mice (Fig. 2l), indicating that PGE2 regulated EP2 expression.

### COX-1 deletion slows down photoreceptor degeneration in rd10 mice

**Fig. 3:**
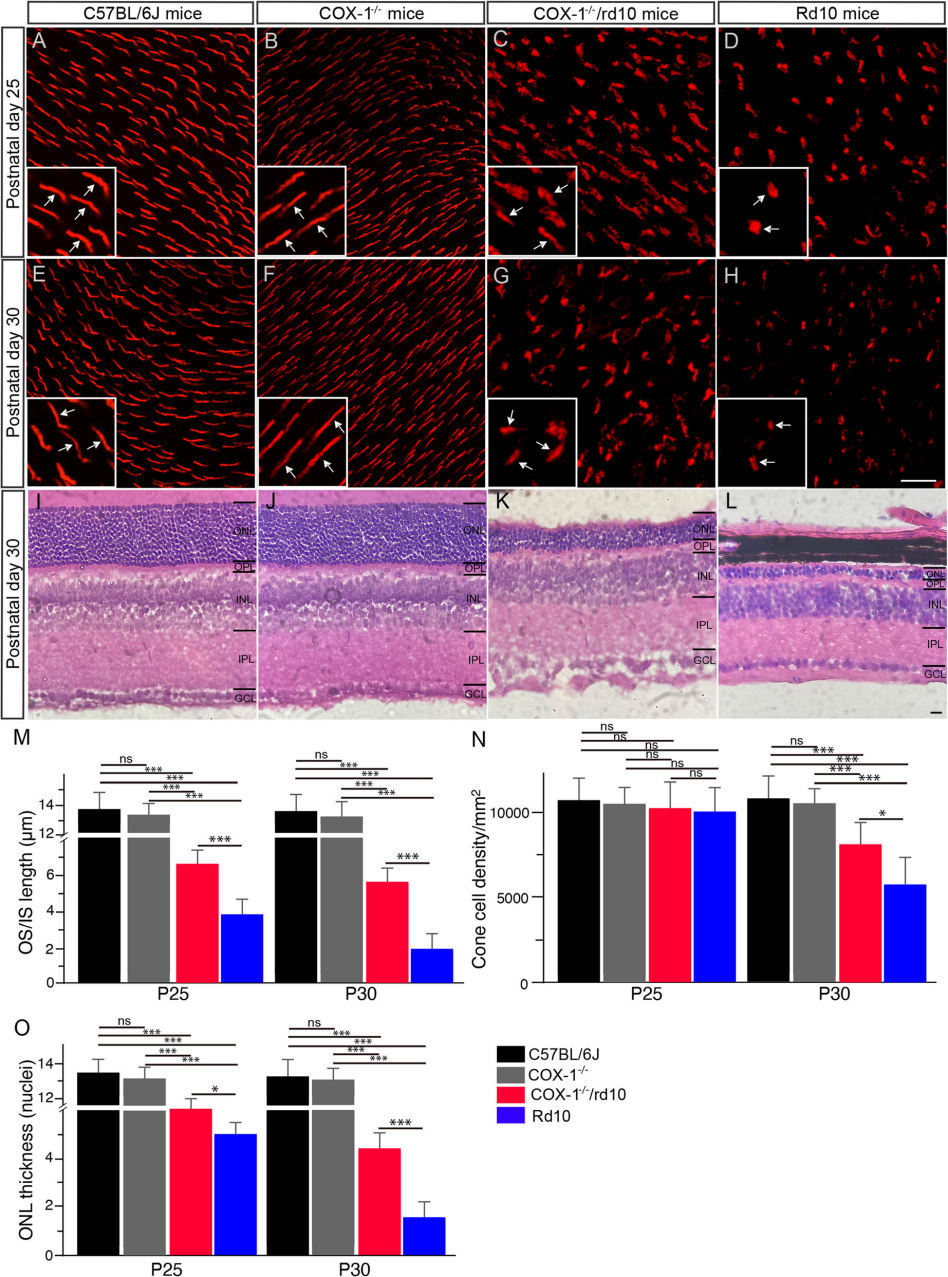
COX-1 deletion preserves photoreceptor morphology in rd10 mice. **a**–**h** Retinal flat mounts were stained with an antibody against red/green opsins. Retinas from the dorsal retina at about 1 mm away from the optic nerve head along the dorsal-ventral axis of WT mice show red/green-opsin-expressing cone outer segment (OS) (**a**, **e**, arrows in insets). Similarly, elongated cone OSs were shown in P25 and P30 COX-1^−/−^ mouse retinas (**b**, **f**, arrows in insets). Cone OS and inner segment (IS) in the retina of P25 and P30 rd10 mice were revealed by the antibody showing that cone OS and IS became flattened (**d**, **h**, arrows in insets), whereas cone OS and IS remained partially intact in age-matched COX-1^−/−^/rd10 mice (**c**, **g**, arrows in insets). **i**–**l** Retinal sections from the dorsal retina at about 1 mm away from the optic nerve head along the dorsal-ventral axis of P30 C57BL/6J (**i**), COX-1^−/−^ (**j**), COX-1^−/−^/rd10 (**k**), and rd10 (**l**) mice were stained with hematoxylin and eosin (H&E). *ONL* outer nuclear layer, *OPL* outer plexiform layer, *INL* inner nuclear layer, *IPL* inner plexiform layer, *GCL* ganglion cell layer. Scale bar, 20 μm. **m** Plot of the average length of cone OS/IS, measured in retinal vertical sections of three groups of mice at P25 (*F*(3, 20) = 23.12, *p* = 1.0 × 10^−6^; WT versus COX-1^−/−^, *p* = 0.45; WT versus rd10, *p* = 2.3 × 10^−5^; WT versus COX-1^−/−^/rd10, *p* = 0.00058; COX-1^−/−^ versus rd10, *p* = 4.0 × 10^−5^; COX-1^−/−^ versus COX-1^−/−^/rd10, *p* = 0.0019; rd10 versus COX-1^−/−^/rd10, *p* = 0.00016), and P30 (*F*(3, 20) = 45.52, *p* = 3.3 × 10^−9^; WT versus COX-1^−/−^, *p* = 0.28; WT versus rd10, *p* = 1.3 × 10^−6^; WT versus COX-1^−/−^/rd10, *p* = 2.6 × 10^−5^; COX-1^−/−^ versus rd10, *p* = 1.7 × 10^−6^; COX-1^−/−^ versus COX-1^−/−^/rd10, *p* = 6.7 × 10^−5^; rd10 versus COX-1^−/−^/rd10, *p* = 0.00061). **n** Quantification of cone cell densities from whole mounted retinas of WT, rd10, and COX-1^−/−^/rd10 mice at P25 (*F*(3, 20) = 0.98, *p* = 0.42; WT versus COX-1^−/−^, *p* = 0.39; WT versus rd10, *p* = 0.066; WT versus COX-1^−/−^/rd10, *p* = 0.15; COX-1^−/−^ versus rd10, *p* = 0.41; COX-1^−/−^ versus COX-1^−/−^/rd10, *p* = 0.75; rd10 versus COX-1^−/−^/rd10, *p* = 0.33), and P30 (*F*(3, 20) = 27.62, *p* = 2.6 × 10^−7^; WT versus COX-1^−/−^, *p* = 0.53; WT versus rd10, *p* = 1.2 × 10^−5^; WT versus COX-1^−/−^/rd10, *p* = 0.0002; COX-1^−/−^ versus rd10, *p* = 4.5 × 10^−5^; COX-1^−/−^ versus COX-1^−/−^/rd10, *p* = 0.0008; rd10 versus COX-1^−/−^/rd10, *p* = 0.012). **o** Plot of the thickness of the outer nuclear layer (ONL), measured in numbers of photoreceptor nuclear row per column in retinal vertical sections of three groups of mice at P25 (*F*(3, 20) = 65.62, *p* = 1.6 × 10^−10^; WT versus COX-1^−/−^, *p* = 0.62; WT versus rd10, *p* = 6.7 × 10^−7^; WT versus COX-1^−/−^/rd10, *p* = 1.6 × 10^−6^; COX-1^−/−^ versus rd10, *p* = 1.1 × 10^−6^; COX-1^−/−^ versus COX-1^−/−^/rd10, *p* = 2.5 × 10^−6^; rd10 versus COX-1^−/−^/rd10, *p* = 0.014), and P30 (*F*(3, 20) = 55.14, *p* = 7.5 × 10^−10^; WT versus COX-1^−/−^, *p* = 0.69; WT versus rd10, *p* = 4.1 × 10^−7^; WT versus COX-1^−/−^/rd10, *p* = 5.9 × 10^−6^; COX-1^−/−^ versus rd10, *p* = 1.8 × 10^−6^; COX-1^−/−^ versus COX-1^−/−^/rd10, *p* = 2.9 × 10^−5^; rd10 versus COX-1^−/−^/rd10, *p* = 1.9 × 10^−5^). Results are presented as means ± SDs (*n* = 6 animals/each group). ns, not significant, *p < 0.05 and ***p < 0.001

We assessed the effect of COX-1 deletion on photoreceptor degeneration in the COX-1^−/−^/rd10 mouse retina. Cone degeneration starts from the initial shortening of the outer segment (OS) followed by the inner segment (IS) in RP retinas [27]. Therefore, we labeled cones with an antibody against red/green opsins and assessed morphological changes in cones in COX-1^−/−^/rd10 mouse retinas. We took sampling areas along the dorsal-ventral axis of retinal whole-mounts, 1 mm from the optic nerve on both sides (Supplementary Figure 2). Red/green-opsin-expressing cone OSs were revealed in WT mouse retinas (Fig. 3a, e, arrows in insets). In COX-1^−/−^ mice, in which COX-1 protein was clearly deleted from the retina (Supplementary Figure 3), we observed normal and elongated cone OSs revealed by the antibody (Fig. 3b, f, arrows in insets). Notably, we observed that cone OS and IS morphology was disrupted in rd10 mice (Fig. 3d, h; arrows in insets), whereas cone OS and IS morphology was largely maintained in age-matched COX-1^−/−^/rd10 mice (Fig. 3c, g; arrows in insets). Measurement of cone OS and IS length on retinal vertical sections showed that up to approximately 50% and 42% of the normal OS length was maintained in P25 and P30 COX-1^−/−^/rd10 retina, respectively (Fig. 3m, red bars), whereas less than about 28% and 14% of the normal OS length remained in P25 and P30 rd10 controls, respectively (Fig. 3m, blue bars). In addition, we quantified cone cell density in retinal flat mounts. We found that cone cell densities were similar among three groups of mice at P25 (Fig. 3n). However, we observed much higher cone densities in P30 COX-1^−/−^/rd10 mice than that in age-matched rd10 retinas (Fig. 3n), indicating the preservation of cones after COX-1 deletion. About 97% of photoreceptor nuclei in the outer nuclear layer (ONL) of the mouse retina belong to rods [28]. Therefore, we measured the ONL thickness in retinal vertical sections for rod survival. We found a higher number of nuclear rows in the ONL revealed by hematoxylin and eosin (H&E) staining in P25 COX-1^−/−^/rd10 mouse retinas than in age-matched rd10 control retinas (Fig. 3o). The similar trend was observed at P30 between COX-1^−/−^/rd10 retinas and rd10 control retinas (Fig. 3k, o), indicating the preservation of rods after COX-1 deletion. Together, our data demonstrated the morphological preservation of rod and cone photoreceptors in the rd10 retina with COX-1 deletion and confirmed the pathological role of COX-1 in the rd10 retina.

### COX-1 deletion preserves retinal function and visual performance in rd10 mice

**Fig. 4:**
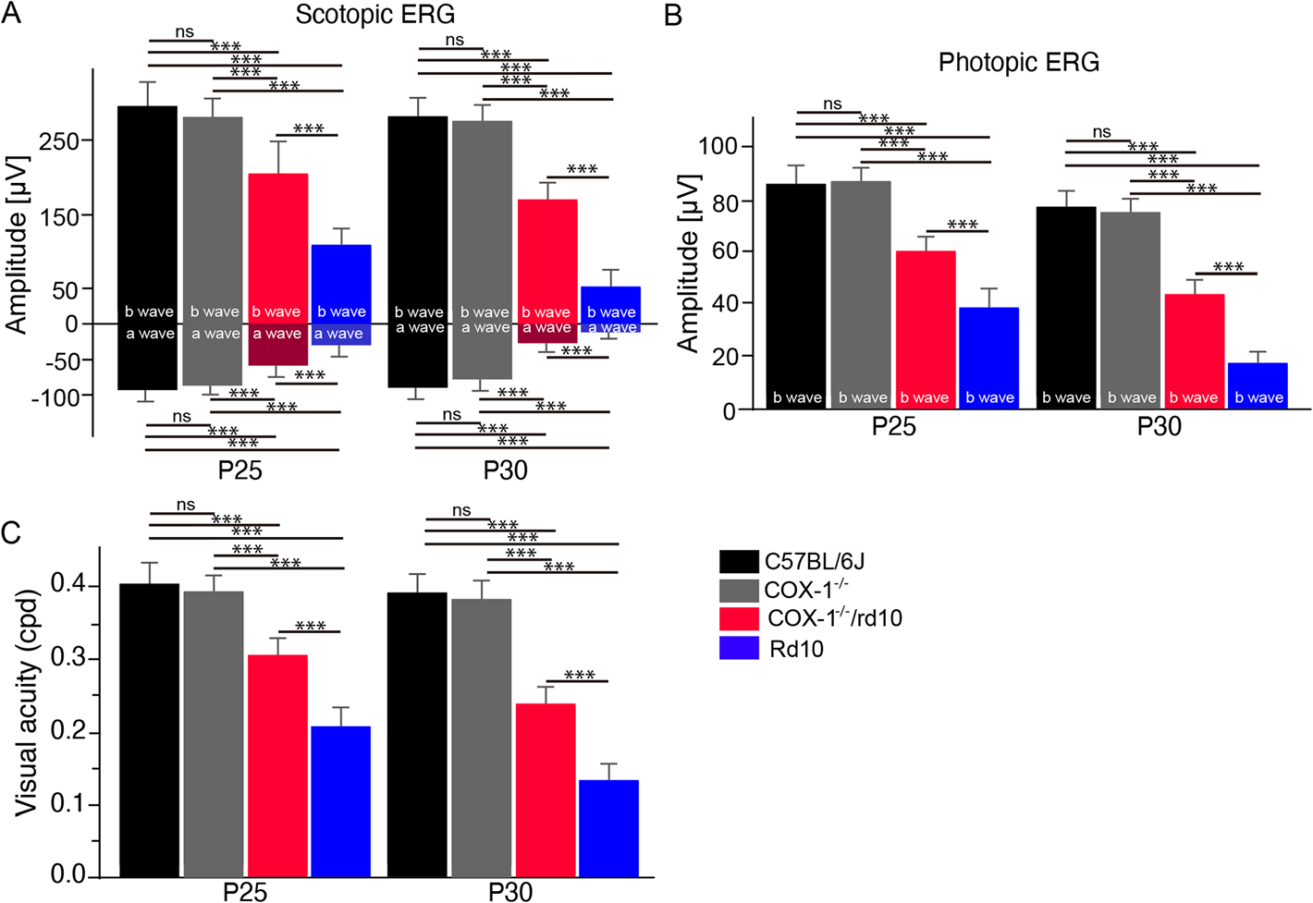
COX-1 deletion preserves retinal function and visual performance in rd10 mice. **a** Statistical analysis of ERG amplitudes. Average scotopic a-wave and b-wave amplitudes elicited at 3 cd-s/m^2^ light intensity from WT, COX-1^−/−^, rd10, and COX-1^−/−^/rd10 mice at P25 (a wave: *F*(3, 20) = 47.79, *p* = 2.6 × 10^−9^; WT versus COX-1^−/−^, *p* = 0.66; WT versus rd10, *p* = 2.7 × 10^−6^; WT versus COX-1^−/−^/rd10, *p* = 0.00013; COX-1^−/−^ versus rd10, *p* = 4.2 × 10^−7^; COX-1^−/−^ versus COX-1^−/−^/rd10, *p* = 2.9 × 10^−5^; rd10 versus COX-1^−/−^/rd10, *p* = 0.00013. b wave: *F*(3, 20) = 52.58, *p* = 1.1 × 10^−9^; WT versus COX-1^−/−^, *p* = 0.99; WT versus rd10, *p* = 1.6 × 10^−6^; WT versus COX-1^−/−^/rd10, *p* = 0.00078; COX-1^−/−^ versus rd10, *p* = 4.4 × 10^−8^; COX-1^−/−^ versus COX-1^−/−^/rd10, *p* = 7.6 × 10^−5^; rd10 versus COX-1^−/−^/rd10, *p* = 1.7 × 10^−5^), and P30 (a wave: *F*(3, 20) = 192.48, *p* = 6.4 × 10^−15^; WT versus COX-1^−/−^, *p* = 0.21; WT versus rd10, *p* = 2.9 × 10^−9^; WT versus COX-1^−/−^/rd10, *p* = 6.0 × 10^−8^; COX-1^−/−^ versus rd10, *p* = 5.2 × 10^−10^; COX-1^−/−^ versus COX-1^−/−^/rd10, *p* = 3.0 × 10^−8^; rd10 versus COX-1^−/−^/rd10, *p* = 1.5 × 10^−5^. b wave: *F*(3, 20) = 167.63, *p* = 2.4 × 10^−14^; WT versus COX-1^−/−^, *p* = 0.98; WT versus rd10, *p* = 7.9 × 10^−9^; WT versus COX-1^−/−^/rd10, *p* = 3.6 × 10^−6^; COX-1^−/−^ versus rd10, *p* = 4.6 × 10^−10^; COX-1^−/−^ versus COX-1^−/−^/rd10, *p* = 1.8 × 10^−7^; rd10 versus COX-1^−/−^/rd10, *p* = 2.6 × 10^−7^). **b** Average photopic b-wave amplitudes elicited at 3 cd-s/m^2^ light intensity on P25 (*F*(3, 20) = 66.41, *p* = 1.4 × 10^-10^; WT versus COX-1^−/−^, *p* = 0.29; WT versus rd10, *p* = 1.6 × 10^-7^; WT versus COX-1^−/−^/rd10, *p* = 2.1 × 10^−5^; COX-1^−/−^ versus rd10, *p* = 6.5 × 10^−7^; COX-1^−/−^ versus COX-1^−/−^/rd10, *p* = 0.00015; rd10 versus COX-1^−/−^/rd10, *p* = 0.00013), and P30 WT, rd10 and COX-1^−/−^/rd10 mice (*F* (3, 20) = 226.35, *p* = 1.3 × 10^−15^; WT versus COX-1^−/−^, *p* = 0.43; WT versus rd10, *p* = 3.0 × 10^−10^; WT versus COX-1^−/−^/rd10, *p* = 1.7 × 10^−7^; COX-1^−/−^ versus rd10, *p* = 2.5 × 10^−10^; COX-1^−/−^ versus COX-1^−/−^/rd10, *p* = 2.6 × 10^−7^; rd10 versus COX-1^−/−^/rd10, *p* = 9.2 × 10^−7^). **c** Photopic visual acuity was evaluated from WT, COX-1^−/−^/, rd10 and COX-1^−/−^/rd10 mice at P25 (*F*(3, 20) = 88.99, *p* = 9.6 × 10^−12^; WT versus COX-1^−/−^, *p* = 0.14; WT versus rd10, *p* = 6.1 × 10^−8^; WT versus COX-1^−/−^/rd10, *p* = 2.0 × 10^−5^; COX-1^−/−^ versus rd10, *p* = 7.2 × 10^−8^; COX-1^−/−^ versus COX-1^−/−^/rd10, *p* = 5.9 × 10^−5^; rd10 versus COX-1^−/−^/rd10, *p* = 1.1 × 10^−5^), and P30 (*F*(3, 20) = 268.55, *p* = 2.5 × 10^−16^; WT versus COX-1^−/−^, *p* = 0.41; WT versus rd10, *p* = 4.4 × 10^−10^; WT versus COX-1^−/−^/rd10, *p* = 6.0 × 10^−8^; COX-1^−/−^ versus rd10, *p* = 2.2 × 10^−10^; COX-1^−/−^ versus COX-1^−/−^/rd10, *p* = 2.4 × 10^−8^; rd10 versus COX-1^−/−^/rd10, *p* = 1.1 × 10^v6^). Results are presented as means ± SDs (*n* = 6 animals/each group). ***p < 0.001

We next assessed the effect of COX-1 deletion on photoreceptor function by measuring electroretinography (ERG) and optomotor responses on COX-1^−/−^/rd10 mice. Markedly greater amplitudes of scotopic a-waves and b-waves were observed on P25 and P30 COX-1^−/−^/rd10 mice relative to age-matched rd10 and COX-1^−/−^/ controls (Fig. 4a). Similarly, higher photopic ERG b-wave amplitudes were recorded on COX-1^−/−^/rd10 mice at the two time points in comparison with age-matched rd10 and COX-1^−/−^/ controls (Fig. 4b). Our data suggested that COX-1 deletion preserved photoreceptor function in rd10 mice. To evaluate spatial visual performance, we measured optomotor responses of COX-1^−/−^/rd10 mice to moving gratings. We found that COX-1^−/−^/rd10 mouse eyes displayed significantly better optomotor responses than age-matched rd10 mouse eyes at P25 and P30 (Fig. 4c), indicating the preservation of visual acuity in rd10 mice with COX-1 deficiency. Together, our data demonstrated the preservation of retinal function and visual performance in rd10 mice with COX-1 deletion

### COX-1 deletion provides long-term preservation of the morphology and function of cone photoreceptors in the rd10 retina

**Fig. 5:**
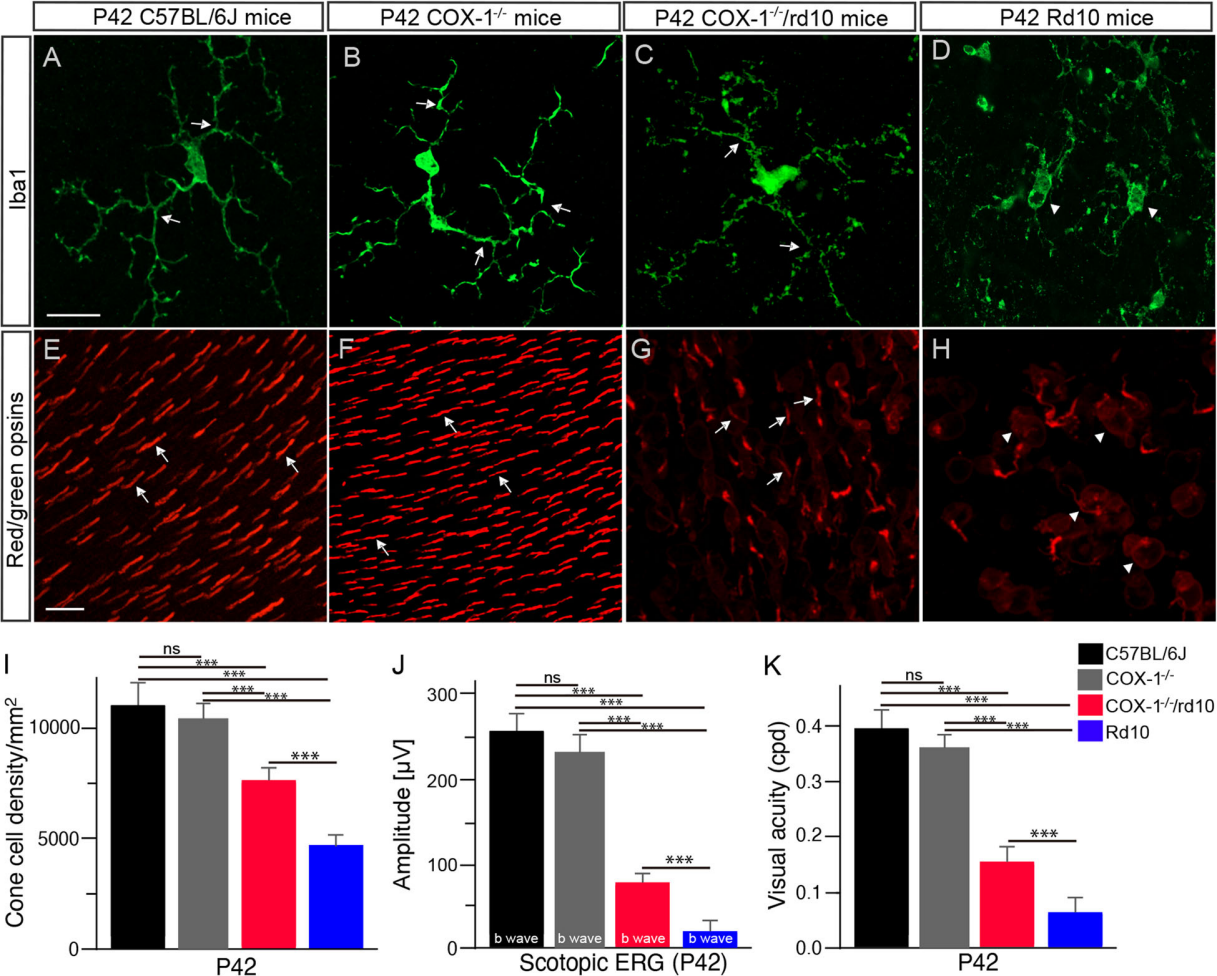
COX-1 deletion provides the long-term inhibition of microglia activation and the preservation of cone morphology and function in the rd10 retina. **a**–**d** Retina flat mounts were stained with an antibody against Iba-1, a marker for microglia. Confocal images from the dorsal retina at about 1 mm away from the optic nerve head along the dorsal-ventral axis show that microglia display a ramified morphology in P42 WT (**a**, arrows) COX-1^−/−^ (**b**, arrows) and COX-1^−/−^/rd10 (**c**, arrows) mouse retinas and an amoeboid appearance in P42 rd10 control retinas (**d**, arrowheads). Scale bar, 10 μm. **e**–**h** Retinal flat mounts were stained with an antibody against red/green opsins. The retina from WT (**e**, arrows) and COX-1^−/−^ (**f**, arrows) mice shows red/green-opsin-expressing cone OS. The antibody reveals shorten cone OS and IS in P42 COX-1^−/−^/rd10 mice (**g**, arrows), while most cone cells lost OS and IS in P42 rd10 control mice (**h**, arrowheads). Scale bar, 20 μm. **i** Quantification of cone cell densities from whole mounted retinas of P42 WT, COX-1^−/−^, rd10 and COX-1^−/−^/rd10 mice (*F*(3, 20) = 76.96, *p* = 3.7 × 10^−11^; WT versus COX-1^−/−^, *p* = 0.43; WT versus rd10, *p* = 1.9 × 10^-7^; WT versus COX-1^−/−^/rd10, *p* = 2.4 × 10^−5^; COX-1^−/−^ versus rd10, *p* = 4.7 × 10^−8^; COX-1^−/−^ versus COX-1^−/−^/rd10, *p* = 1.3 × 10^−5^; rd10 versus COX-1^−/−^/rd10, *p* = 1.5 × 10^−11^). **j** Average scotopic b-wave amplitudes in response to 3 cd-s/m^2^ flash light intensity (*F* (3, 20) = 946.87, *p* = 1.0 × 10^−21^; WT versus COX-1^−/−^, *p* = 0.64; WT versus rd10, *p* = 1.4 × 10^−13^; WT versus COX-1^−/−^/rd10, *p* = 3.6 × 10^−1^; COX-1^−/−^ versus rd10, *p* = 2.7 × 10^−13^; COX-1^−/−^ versus COX-1^−/−^/rd10, *p* = 3.7 × 10^−12^; rd10 versus COX-1^−/−^/rd10, *p* = 4.2 × 10^−12^). **k** Photopic visual acuity was evaluated from P42 WT, COX-1^−/−^, rd10, and COX-1^−/−^/rd10 mice (*F*(3, 20) = 785.53, *p* = 6.6 × 10^−21^; WT versus COX-1^−/−^, *p* = 0.23; WT versus rd10, *p* = 5.1 × 10^−12^; WT versus COX-1^−/−^/rd10, *p* = 1.3 × 10^−10^; COX-1^−/−^ versus rd10, *p* = 6.8 × 10^−13^; COX-1^−/−^ versus COX-1^−/−^/rd10, *p* = 1.6 × 10^−11^; rd10 versus COX-1^−/−^/rd10, *p* = 1.1 × 10^−7^). Results are presented as means ± SDs (*n* = 6 animals/each group). ***p < 0.001

We investigated the long-term effect of COX-1 deletion on microglia activation and photoreceptor degeneration in the rd10 retina. Microglia display a ramified morphology in P42 WT mouse retinas (Fig. 5a, arrows). Microglia in the P42 rd10 retina presented a thicker microglial cell body area with a reduced branching pattern and an uneven distribution of the branching (Fig. 5c, arrowheads) compared to microglia in age-matched WT mice (Fig. 5a, arrows). However, microglia maintained a ramified appearance in P42 COX-1^−/−^/rd10 mouse retinas (Fig. 5b, arrows), indicating the long-term suppression of microglia activation in the rd10 mouse retina with COX-1 deletion. The antibody against red/green opsins revealed red/green-opsin-expressing cone OS in WT mouse retinas (Fig. 5d, arrowheads), and the loss of cone OS and IS in rd10 mice (Fig. 5f, arrowheads). Meanwhile, cone cells still maintained elongated OS and IS morphology in P42 COX-1^−/−^/rd10 mouse retinas (Fig. 5e, arrows), indicating the long-term morphological preservation of cone cells after COX-1 deletion. Moreover, COX-1 deletion improved cone cell survival rate (Fig. 5g, red bar), scotopic b-wave amplitudes (Fig. 5h, red bar), and visual acuity (Fig. 5i, red bar) in P42 rd10 mice. Together, our data showed the long-term suppression of microglia activation and the preservation of cone morphology and function in the rd10 retina with COX-1 deletion.

### The inflammatory PGE2 EP2 receptor is the downstream effector of COX-1-mediated neurotoxicity in rd10 mice

**Fig. 6:**
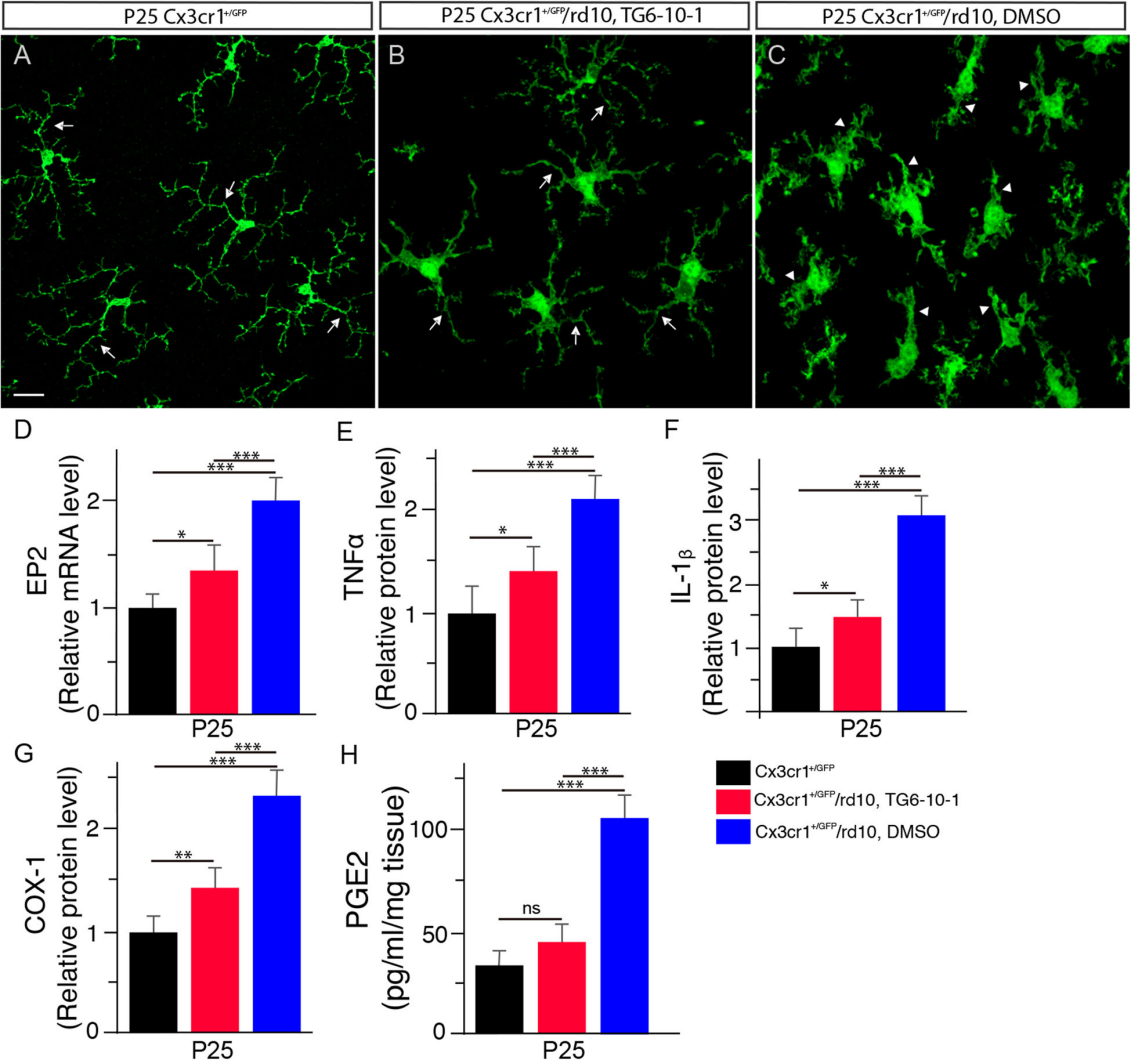
Inhibition of the prostaglandin receptor EP2 by TG6-10-1 reduces microgliosis in rd10 mice. Confocal images from the dorsal retina at about 1 mm away from the optic nerve head along the dorsal-ventral axis show that microglia display a ramified morphology in P25 WT (**a**, arrows). Microglia maintained a ramified appearance in TG6-10-1-treated rd10 retinas (**b**, arrows), whereas microglia show an amoeboid morphology in DMSO-treated rd10 controls (**c**, arrowheads). Scale bar: 20 μm. **d** qPCR analyses of EP2 mRNA levels in retinal microglial cells sorted by FACS from P25 CX3CR1^+/GFP^ and rd10/CX3CR1^+/GFP^ mice treated with TG6-10-1 or DMSO (*F*(2, 15) = 32.61, *p* = 3.5 × 10^−6^; WT versus DMSO-treated rd10, *p* = 6.4 × 10^−6^; WT versus TG6-10-1 treated rd10, *p* = 0.027; DMSO-treated rd10 versus TG6-10-1 treated rd10, *p* = 0.00016). **e** ELISA analyses of TNF-α expression levels in the retina of three groups (*F* (2, 15) = 33.19, *p* = 3.1 × 10^−6^; WT versus DMSO- treated rd10, *p* = 4.7 × 10^−6^; WT versus TG6-10-1 treated rd10, *p* = 0.011; DMSO-treated rd10 versus TG6-10-1 treated rd10, *p* = 0.00036). **f** ELISA analyses of IL-1β expression levels in the retina of three groups (*F*(2, 15) = 210.37, *p* = 1.1 × 10^−11^; WT versus DMSO- treated rd10, *p* = 8.9 × 10^−10^; WT versus TG6-10-1 treated rd10, *p* = 0.015; DMSO-treated rd10 versus TG6-10-1 treated rd10, *p* = 2.5 × 10^-8^). **g** ELISA analyses of COX-1 expression levels in the retina of three groups (*F*(2, 15) = 56.53, *p* = 1.1 × 10^−7^; WT versus DMSO-treated rd10, *p* = 1.9 × 10^−7^; WT versus TG6-10-1 treated rd10, *p* = 0.0071; DMSO-treated rd10 versus TG6-10-1 treated rd10, *p* = 3.6 × 10^−5^). **h** ELISA analyses of PGE2 expression levels in the retina of three groups (*F*(2, 15) = 96.95, *p* = 2.6 × 10^−9^; WT versus DMSO-treated rd10, *p* = 3.1 × 10^−7^; WT versus TG6-10-1 treated rd10, *p* = 0.058; DMSO-treated rd10 versus TG6-10-1 treated rd10, *p* = 5.5 × 10^−7^). Results are presented as means ± SDs (*n* = 6 animals/each group). *ns* not significant, *p < 0.05, **p < 0.01, and ***p < 0.001

We next sought to identify whether PGE2 signaling through EP2 receptors was involved in regulating inflammatory responses in the rd10 retina. For this reason, we administered rd10 or rd10/CX3CR1^+/GFP^ mice with TG6-10-1, a specific EP2 receptor antagonist [10]. We found that systemic administration of TG6-10-1 largely recapitulated the beneficial effects of COX-1 ablation in the rd10 retina. Microglia maintained a ramified morphology in P25 rd10/CX3CR1^+/GFP^ mouse retinas after TG6-10-1 treatment (Fig. 6b, arrows), while microglia displayed an amoeboid shape in age-matched rd10/CX3CR1^+/GFP^ retinas treated with DMSO (Fig. 6c, arrowheads). In addition, we found that EP2 transcripts in sorted primary microglial cells were downregulated in the rd10 retina after TG6-10-1 treatment (Fig. 6d), confirming EP2 inhibition in microglial cells after the drug treatment. Moreover, ELISA quantification revealed reduced expressions of TNF-α (Fig. 6e) and IL-1β (Fig. 6f) proteins, indicating reductions in the production of these proinflammatory molecules after TG6-10-1 treatment. Interestingly, we also observed reduced protein expressions of COX-1 (Fig. 6g) and PGE2 (Fig. 6h) after EP2 inhibition by TG6-10-1, which further supported a proinflammatory function for COX-1.

**Fig. 7:**
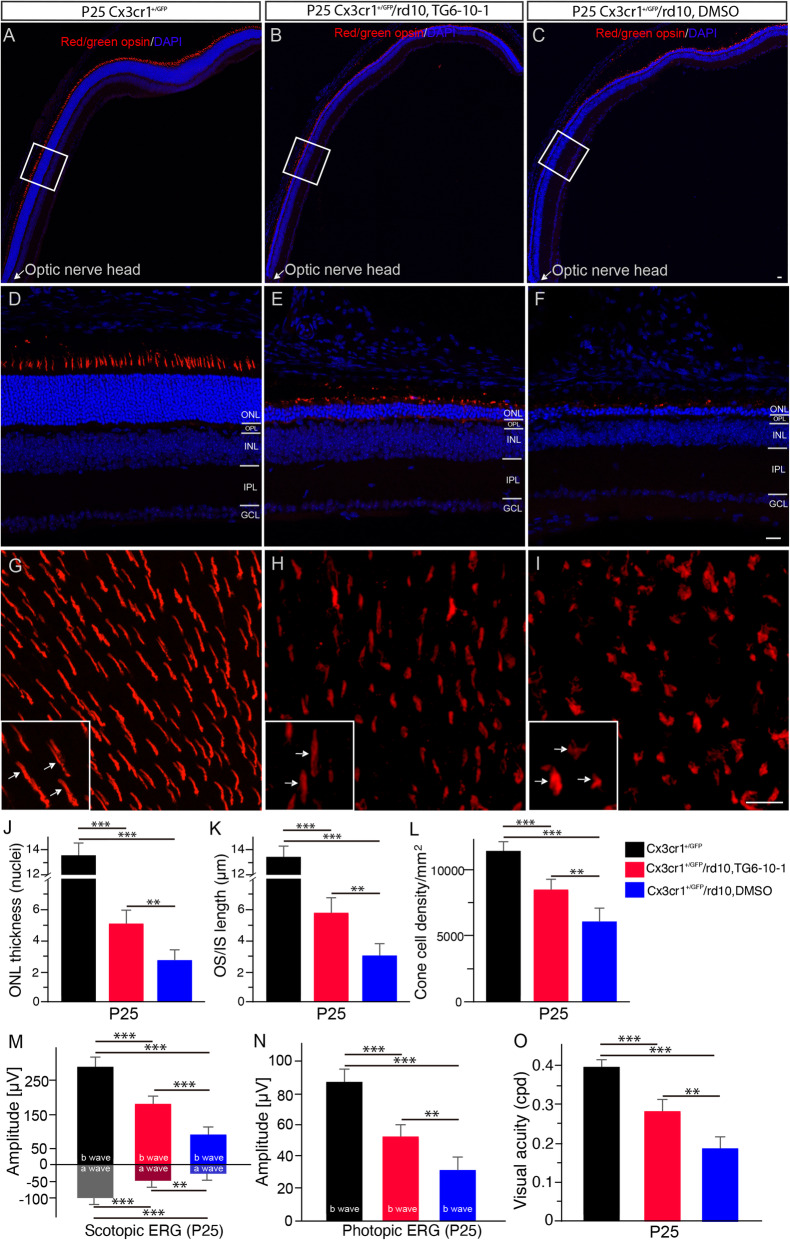
Inhibition of the prostaglandin receptor EP2 by TG6-10-1 preserves photoreceptors in rd10 mice. **a**–**c** A long stretch of a retinal section from P25 CX3CR1^+/GFP^ (A) and rd10/CX3CR1^+/GFP^ mice treated with TG6-10-1 (**b**) or DMSO (**c**) were stained an antibody against red/green opsins showing cone OS in WT mouse retinas (**a**, red) and cone OS and IS in rd10 mouse retinas (**b**, **c**, red). Cell nuclei were stained with DAPI (blue). **d**–**f** Retinal sections from the boxed regions in **a**, **b**, and **c**. *ONL* outer nuclear layer, *OPL* outer plexiform layer, *INL* inner nuclear layer, *IPL* inner plexiform layer, *GCL* ganglion cell layer. **g**–**i** Retinal flat from the dorsal retina at about 1 mm away from the optic nerve head along the dorsal-ventral axis of P25 CX3CR1^+/GFP^ (**g**) and rd10/CX3CR1^+/GFP^ mice treated with TG6-10-1 (**h**) or DMSO (**i**) were stained with an antibody against red/green opsins. The retina from WT mice shows red/green-opsin-expressing cone OSs (**g**, arrows in inset). Cone OS and IS in the retina of P25 rd10/CX3CR1^+/GFP^ mice treated with TG6-10-1 remained partially intact (**h,** arrows in inset), whereas cone OS and IS in the retina of P25 rd10/CX3CR1^+/GFP^ mice treated with DMSO were flattened (**i**, arrows in inset). **j** Average thickness of the ONL, measured in numbers of photoreceptor nuclear rows per column in retinal vertical sections (*F*(2, 15) = 160.69, *p* = 7.4 × 10^−11^; WT versus DMSO-treated rd10, *p* = 5.8 × 10^−9^; WT versus TG6-10-1 treated rd10, *p* = 1.2 × 10^−7^; DMSO-treated rd10 versus TG6-10-1 treated rd10, *p* = 0.0016). **k** Average length of cone OS/IS, measured in retinal vertical sections (*F*(2, 15) = 51.88, *p* = 1.8 × 10^−7^; WT versus DMSO-treated rd10, *p* = 2.6 × 10^−6^; WT versus TG6-10-1 treated rd10, *p* = 4.6 × 10^−5^; DMSO-treated rd10 versus TG6-10-1 treated rd10, *p* = 0.0015). Scale bars: 20 μm. **l** Quantification of cone cell densities from flat mounted retinas of P25 CX3CR1^+/GFP^ and rd10/CX3CR1^+/GFP^ mice treated with TG6-10-1 or DMSO (*F*(2, 15) = 78.82, *p* = 1.1 × 10^−8^; WT versus DMSO-treated rd10, *p* = 1.8 × 10^−7^; WT versus TG6-10-1 treated rd10, *p* = 9.8 × 10^−7^; DMSO-treated rd10 versus TG6-10-1 treated rd10, *p* = 0.0017). **m** Scotopic ERG responses to 3 cd-s/m^2^ light intensity from P25 CX3CR1^+/GFP^ WT and rd10/CX3CR1^+/GFP^ mice treated with TG6-10-1 or DMSO. Statistical analysis of scotopic a-wave amplitudes (*F*(2, 15) = 38.21, *p* = 1.3 × 10^−6^; WT versus DMSO-treated rd10, *p* = 1.1 × 10^−5^; WT versus TG6-10-1 treated rd10, *p* = 0.00017; DMSO-treated rd10 versus TG6-10-1 treated rd10, *p* = 0.0015), and b-wave amplitudes (F (2, 15) = 123.23, *p* = 4.9 × 10^−10^; WT versus DMSO-treated rd10, *p* = 7.9 × 10^−9^; WT versus TG6-10-1 treated rd10, *p* = 7.1 × 10^−7^; DMSO-treated rd10 versus TG6-10-1 treated rd10, *p* = 0.00024). **n** Photopic b-wave amplitudes from P25 CX3CR1^+/GFP^ WT and rd10/CX3CR1^+/GFP^ mice treated with TG6-10-1 or DMSO in response to 3 cd-s/m^2^ flash light intensity (*F*(2, 15) = 60.85, *p* = 6.3 × 10^−8^; WT versus DMSO-treated rd10, *p* = 2.9 × 10^−7^; WT versus TG6-10-1 treated rd10, *p* = 2.9 × 10^−5^; DMSO-treated rd10 versus TG6-10-1 treated rd10, *p* = 0.0012). **o** Visual acuity was evaluated from P25 CX3CR1^+/GFP^ WT and rd10/CX3CR1^+/GFP^ mice treated with TG6-10-1 or DMSO (*F*(2, 15) = 69.48, *p* = 2.6 × 10^−8^; WT versus DMSO-treated rd10, *p* = 3.9 × 10^−8^; WT versus TG6-10-1 treated rd10, *p* = 1.4 × 10^−5^; DMSO-treated rd10 versus TG6-10-1 treated rd10, *p* = 0.0011). Results are presented as means ± SDs (*n* = 6 animals/each group). **p < 0.01 and ***p < 0.001

Moreover, we observed that EP2 inhibition by TG6-10-1 slowed down photoreceptor degeneration in rd10 mice (Fig. 7). A long stretch of retinal sections and magnified images from the sections showed the preservation in the number of nuclear rows in the ONL stained by DAPI in P25 rd10 mouse retinas after TG6-10-1 treatment (Fig. 7b, e, blue) in relative to rd10 controls treated with DMSO (Fig. 7c, f, blue). This observation was confirmed by measurements (Fig. 7j). We also found that TG6-10-1 treatment partially preserved the OS and IS length of cone photoreceptors labeled with red/green opsins (Fig. 7e, red) when compared with age-matched DMSO-treated rd10 mice (Fig. 7f, red). The preservation of cone morphology was well revealed in flat mounted retinas (Fig. 7h, arrows in insets). Quantification confirmed the partial preservation of cone OS and IS length after TG6-10-1 treatment (Fig. 7k, red bar). In addition, more cone cells were preserved in the TG6-10-1-treated rd10 retina (Fig. 7h, l) compared with those in age-matched DMSO-treated rd10 mice (Fig. 7i, l).

Furthermore, we found that TG6-10-1 treatment similarly preserved retinal function and visual performance in rd10 mice. Significant higher amplitudes of scotopic a-waves and b-waves were observed in the eyes of TG6-10-1-treated P25 rd10 mice when compared with age-matched rd10 control mice (Fig. 7m), indicating the preservation of rod function. Similarly, cone function was preserved in the eyes of TG6-10-1-treated rd10 mice at P25 as revealed by higher photopic ERG b-wave amplitudes (Fig. 7n) in relative to age-matched rd10 control mice (Fig. 7n). Moreover, visual function was well preserved in TG6-10-1-treated rd10 mice (Fig. 7o) in comparison with age-matched DMSO-treated rd10 mice (Fig. 7o). Collectively, our data demonstrated that EP2 inhibition by TG6-10-1 preserves retinal function and visual performance in rd10 mice.

Taken together, neuroprotection by an EP2 receptor antagonist supported the critical involvement of this key prostaglandin receptor EP2 in neuroinflammation in rd10 retinas. Our findings indicate that the PGE2 EP2 signaling was essential for the neurotoxicity mediated by COX-1-derived PGE2.

**Fig. 8:**
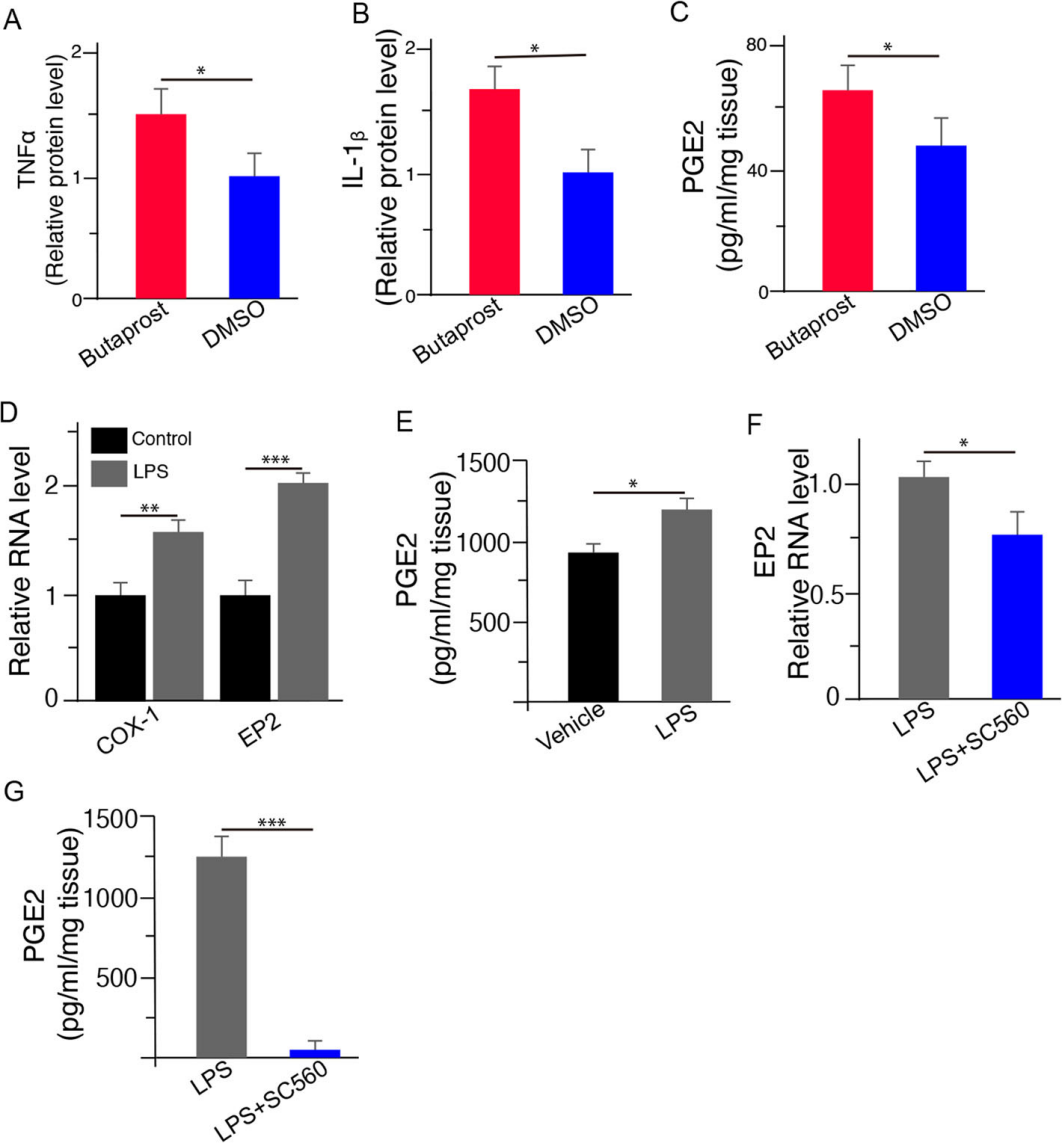
Activation of EP2 receptors by butaprost upregulates pro-inflammatory cytokines in rd10 mice with COX-1 deletion. **a**–**c** Butaprost treatment upregulated the expression levels of pro-inflammatory cytokine TNF-α (**a**, *p* = 0.022, Student *T* test) and IL-1β (**b**, *p* = 0.014, Student *T* test) and PGE2 (**c**, *p* = 0.045, Student *T* test) in the COX-1^−/−^/rd10 mouse retina. **d** LPS stimulation induced COX-1 (*p* = 0.0095, Student *T* test and EP2 (*p* = 0.0009, Student *T* test) expression in BV-2 microglial cells. **e** LPS stimulation induced PGE2 expression in BV-2 microglial cells (*p* = 0.0329, Student *T* test). **f** Elevated EP2 expression induced by LPS stimulation was reduced after pharmacological blockade of COX-1 by the COX-1 inhibitor SC-560 (*p* = 0.0464, Student *T* test). **g** Elevated PGE2 expression level induced by LPS stimulation was reduced after pharmacological blockade of COX-1 by the COX-1 inhibitor SC-560 (*p* < 0.00001, Student *T* test). Values represent the mean ± SD (*n* = 4). *p < 0.05, **p < 0.01 and ***p < 0.0001

To further confirm the involvement of EP2 receptors, we treated COX-1^−/−^/rd10 mice with the EP2 agonist butaprost [29]. We found that butaprost treatment significantly upregulated the expression levels of pro-inflammatory cytokines, including TNF-α (Fig. 8a) and IL-1β (Fig. 8b) in the COX-1^−/−^/rd10 mouse retina. Interestingly, we observed that pharmacological activation of EP2 receptors by butaprost also increased PGE2 expression in the COX-1^−/−^/rd10 mouse retina (Fig. 8c). Moreover, in vitro experiments showed that LPS stimulation increased the expression levels of COX-1, EP2, and PGE2 (Fig. 8d, e). Conversely, the elevated EP2 and PGE2 induced by LPS were reduced after pharmacological blockade of COX-1 by the COX-1 inhibitor SC-560 (Fig. 8f, g). Our data further suggest that the COX-1-PGE2-EP2 signaling was involved in regulation neuroinflammation and neurotoxicity in Rd10 mice.

## Discussion

In this study, we demonstrated the involvement of COX-1 in modulating proinflammatory responses and disease progression in the rd10 mouse model of retinitis pigmentosa (RP). We found that COX-1 deletion or inhibition significantly reduced neuroinflammation and preserved retinal photoreceptor and function and visual performance in rd10 mice. Moreover, we observed that the pharmacological inhibition of the prostaglandin E2 (PGE2) EP2 receptors largely replicated the beneficial effects of COX-1 deletion and delayed photoreceptor degeneration in rd10 mice, suggesting that EP2 receptor was a critical downstream effector of COX-1-mediated neurotoxicity in rd10 mice. Collectively, we demonstrated that the COX-1/PGE2/EP2 signaling pathway is partly responsible for significantly increased neuroinflammation and neurotoxicity in rd10 mice.

Previous studies reported that COX-1 deletion or inhibition reduces inflammatory responses and brain injury [14, 30]. Similarly, COX-1 inhibition is reported to be beneficial in Alzheimer’s disease (AD) patients [16]. Consistent with these previous observations, we found that COX-1 deletion attenuated inflammatory responses and photoreceptor degeneration in rd10 mice, suggesting that COX-1 contributed to neuroinflammation and neurotoxicity in rd10 mice. In addition, we found that COX-1 deletion significantly reduced disease-elevated PGE2 in the rd10 retina and confirmed that disease-induced PGE2 elevation in the rd10 retina was primarily COX-1-dependent in the rd10 retina. Moreover, we explored the downstream PGE2 signaling pathways of COX-1. We found that EP2 receptor blockade using an EP2 inhibitor provided beneficial effects to a similar degree as COX-1 deletion. The anti-inflammatory effects of EP2 inhibition suggested that EP2 receptor activation by PGE2 might underlie COX-1-mediated neurotoxicity in rd10 mice. More importantly, our finding raises the possibility that an EP2 antagonist could be used as a potential therapy to oppose neuroinflammation in RP. Consistent with our findings, EP2 deletion is reported to reduce inflammation and pathology in other neurodegenerative disorders [10–12, 31]. The previous data together with our current findings suggest a general and critical role for EP2 receptor in neuroinflammation and neurodegeneration.

Our data provided clear evidence for a pathological role of the COX-1/PGE2/EP2 signaling pathway in the rd10 retina. Based on our findings, we proposed that elevated COX-1 in the rd10 retina facilitated PGE2 production, which in turn maintained EP2 receptor activation and upregulated inflammatory molecules through EP2 receptor. Evidently, COX-1 deletion led to a significant reduction in PGE2 production (Fig. 2k), which subsequently downregulated EP2 receptor expression (Fig. 2l). Interestingly, we found that pharmacological inhibition of EP2 receptors decreased both COX-1 (Fig. 6g) and PGE2 (Fig. 6h) expression levels in the rd10 retina, conversely suggesting that EP2 receptor activation might induce COX-1 expression to reinforce neuroinflammation in the rd10 retina, which might be an important molecular mechanism of chronic neuroinflammation in RP. Indeed, the activation of EP2 receptors by an EP2 agonist increased the expression levels of pro-inflammatory cytokines as well as PGE2 in the rd10 retina with COX-1 deletion (Fig. 8a–c). On the contrary, either COX-1 deletion or EP2 receptor blockade broke this reinforcing process of the activation and reduced chronic neuroinflammation and subsequently alleviated photoreceptor degeneration in the rd10 retina. Furthermore, our results from in vitro work largely recapitulated findings observed in vivo experiments, further suggesting the involvement of the COX-1/PGE2/EP2 signaling pathway in the rd10 retina.

## Conclusions

In conclusion, we demonstrated the COX-1/PGE2/EP2 signaling pathway as a prominent player in triggering chronic neuroinflammation in rd10 mice. More importantly, we provided a proof of concept that proinflammatory COX-1 signaling in a RP animal model could be suppressed pharmacologically, suggesting a potential path to a practical clinical application from our data. Moreover, our findings on the pathological role of EP2 receptor in rd10 mice might provide a rationale for developing new anti-inflammatory therapeutics targeting EP2 receptor. More selective inhibition of inflammatory EP2 signaling pharmacologically might block the pathological activity of COX-1 without the potential side effects from broader COX-1 inhibition in treating RP patients.

## Supplementary information

Additional file 1: Supplementary Figure 1. Downregulation of pro-inflammatory cytokines and preservation of photoreceptors by COX-1-specific inhibitor SC-560 in rd10 mice. A-C. Confocal images from the dorsal retina at about 1 mm away from the optic nerve head along the dorsal-ventral axis show that microglia display a ramified morphology in P25 CX3CR1^+/GFP^ WT (A, arrows). Microglia maintained a ramified appearance in SC-560-treated CX3CR1^+/GFP^/rd10 mouse retinas (B, arrows), whereas microglia show an amoeboid morphology in DMSO-treated CX3CR1^+/GFP^/rd10 controls (C, arrowheads). Scale bar: 20 μm. D-G. ELISA analyses of TNF-α (D), IL-1β (E), COX-2 (F) and PGE2 (G) show that pro-inflammatory cytokines are downregulated following SC-560 treatment. H. Plot of the thickness of the ONL, measured in numbers of photoreceptor nuclei per column. Results are presented as means ± SDs (n = 5 animals/each group). ns, not significant, * p < 0.05 and *** p < 0.001. (TIF 654 KB)

Additional file 2: Supplementary Figure 2. A lower power confocal image from the dorsal retina of a C57BL/6J mouse shows the outer segments of red/green cone photoreceptors revealed by an antibody agaonst red/green opsins (red). A white square indicates the region, which is about 1mm away from the ONH, is our sampling area from the whole mounted retina. ONH, optic nerve head. Scale bar: 20 μm. (TIF 1.72 MB)

Additional file 3: Supplementary Figure 3. Retinal sections from the dorsal retina at about 1 mm away from the optic nerve head along the dorsal-ventral axis of adult C57BL/6J (A-C) and COX-1^-/-^ (D-F) mice were stained with an anti-rabbit COX-1 antibody (red) and Iba-1 (green), a microglial and macrophage-specific marker. Cell nuclei were stained with 4′,6-diamidino-2-phenylindole (DAPI) (blue). Arrows indicate colocalization between microglia (green) and COX-1 signal (red) in the C57BL/6J mouse retina (A-C), while an arrowhead points to a microglia cell without any COX-1 signal in the COX-1^-/-^ mouse retina (D-F). ONL, outer nuclear layer; OPL, outer plexiform layer; INL, inner nuclear layer; IPL, inner plexiform layer; GCL, ganglion cell layer. Scale bar: 20 μm. (TIF 2.83 MB)

## Abbreviations

COX-1: Cyclooxygenase-1
RP: Retinitis pigmentosa
WT: Wild type
PGE2: Prostaglandin E2
GFP: Green fluorescent protein
PFA: Paraformaldehyde
NGS: Normal goat serum
BSA: Bovine serum albumin
PBS: Phosphate-buffered saline
ERG: Electroretinographic
FACS: Fluorescence-activated cell sorting
ELISA: Enzyme-linked immunosorbent assay
IL-1β: Interleukin-1 beta
TNF-α: Tumor necrosis factor-alpha
OS: Outer segment
IS: Inner segment
ONL: Outer nuclear layer
OPL: Outer plexiform layer
INL: Inner nuclear layer
IPL: Inner plexiform layer
GCL: Ganglion cell layer
