# Supplementary figures and images for: Cyclooxygenase-1 mediates neuroinflammation and neurotoxicity in a mouse model of retinitis pigmentosa
Source: J Neuroinflammation. 2020 Oct 15;17:306. doi: 10.1186/s12974-020-01993-0 (PMC7565369; doi:10.1186/s12974-020-01993-0)

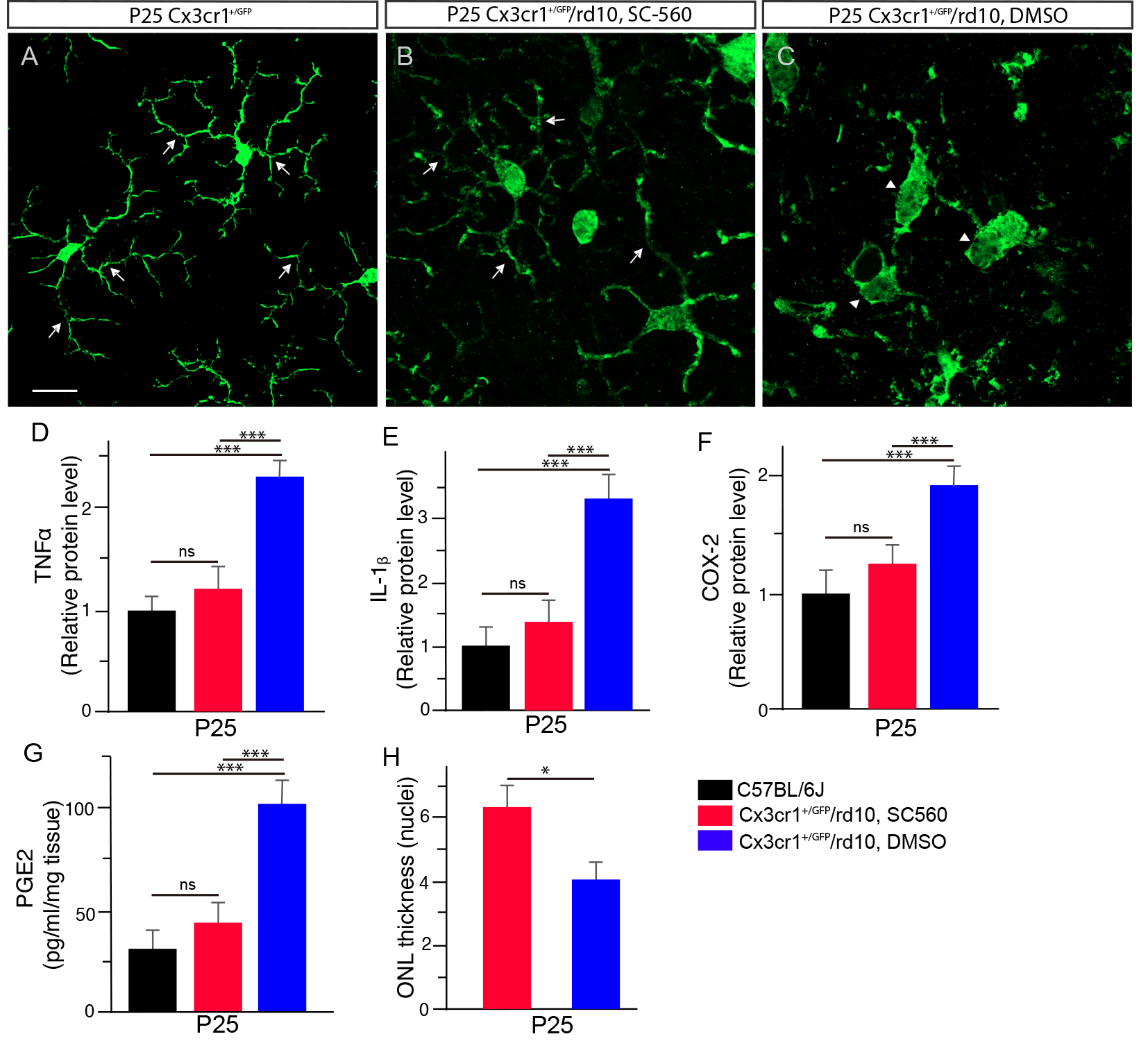

Supplement: Supplementary file 1 — Supplementary Figure 1. Downregulation of pro-inflammatory cytokines and preservation of photoreceptors by COX-1-specific inhibitor SC-560 in rd10 mice. A-C. Confocal images from the dorsal retina at about 1 mm away from the optic nerve head along the dorsal-ventral axis show that microglia display a ramified morphology in P25 CX3CR1+/GFP WT (A, arrows). Microglia maintained a ramified appearance in SC-560-treated CX3CR1+/GFP/rd10 mouse retinas (B, arrows), whereas microglia show an amoeboid morphology in DMSO-treated CX3CR1+/GFP/rd10 controls (C, arrowheads). Scale bar: 20 μm. D-G. ELISA analyses of TNF-α (D), IL-1β (E), COX-2 (F) and PGE2 (G) show that pro-inflammatory cytokines are downregulated following SC-560 treatment. H. Plot of the thickness of the ONL, measured in numbers of photoreceptor nuclei per column. Results are presented as means ± SDs (n = 5 animals/each group). ns, not significant, * p < 0.05 and *** p < 0.001. (TIF 654 KB) [file 12974_2020_1993_MOESM1_ESM.tif]

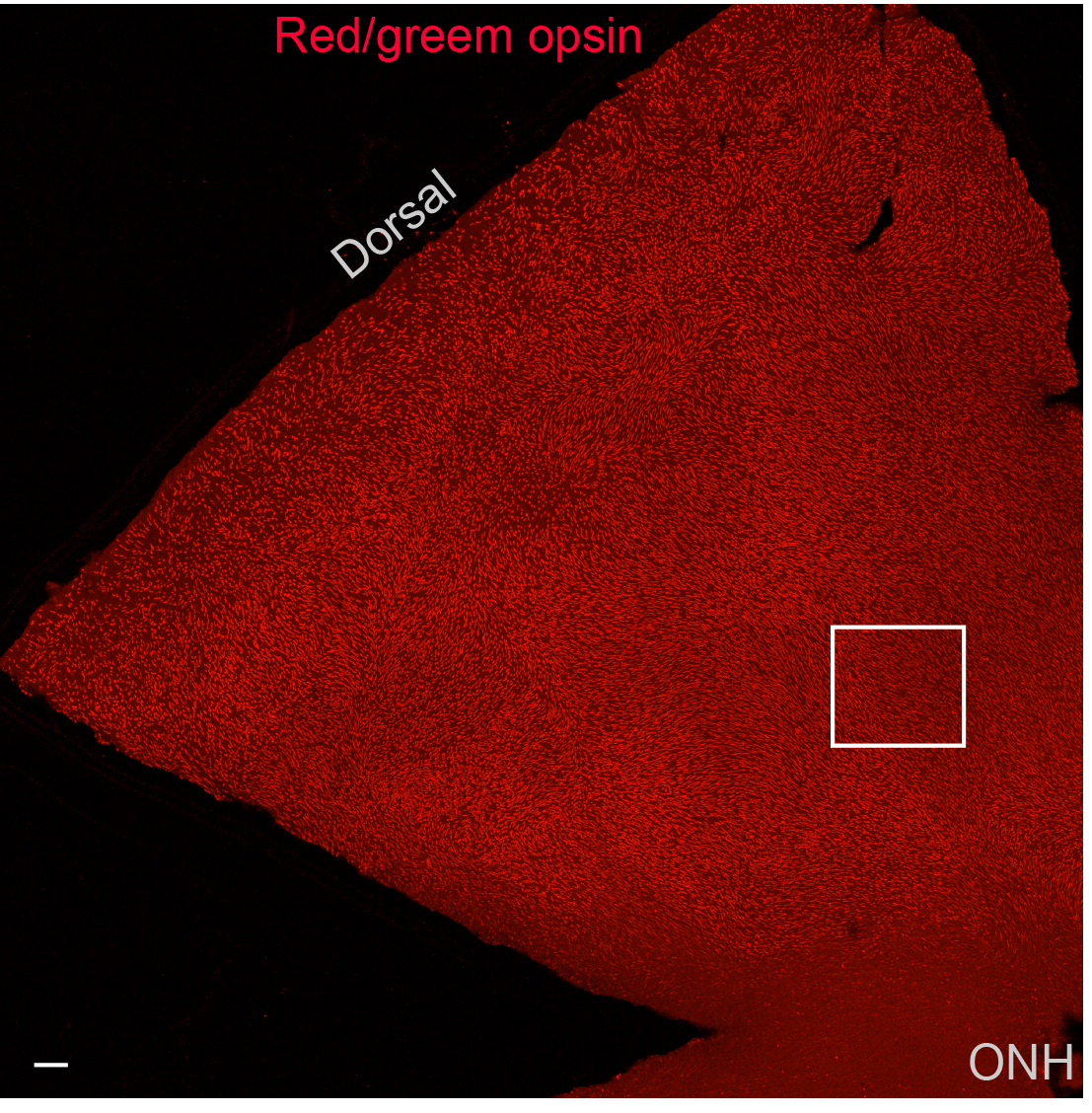

Supplement: Supplementary file 2 — Supplementary Figure 2. A lower power confocal image from the dorsal retina of a C57BL/6J mouse shows the outer segments of red/green cone photoreceptors revealed by an antibody agaonst red/green opsins (red). A white square indicates the region, which is about 1mm away from the ONH, is our sampling area from the whole mounted retina. ONH, optic nerve head. Scale bar: 20 μm. (TIF 1.72 MB) [file 12974_2020_1993_MOESM2_ESM.tif]

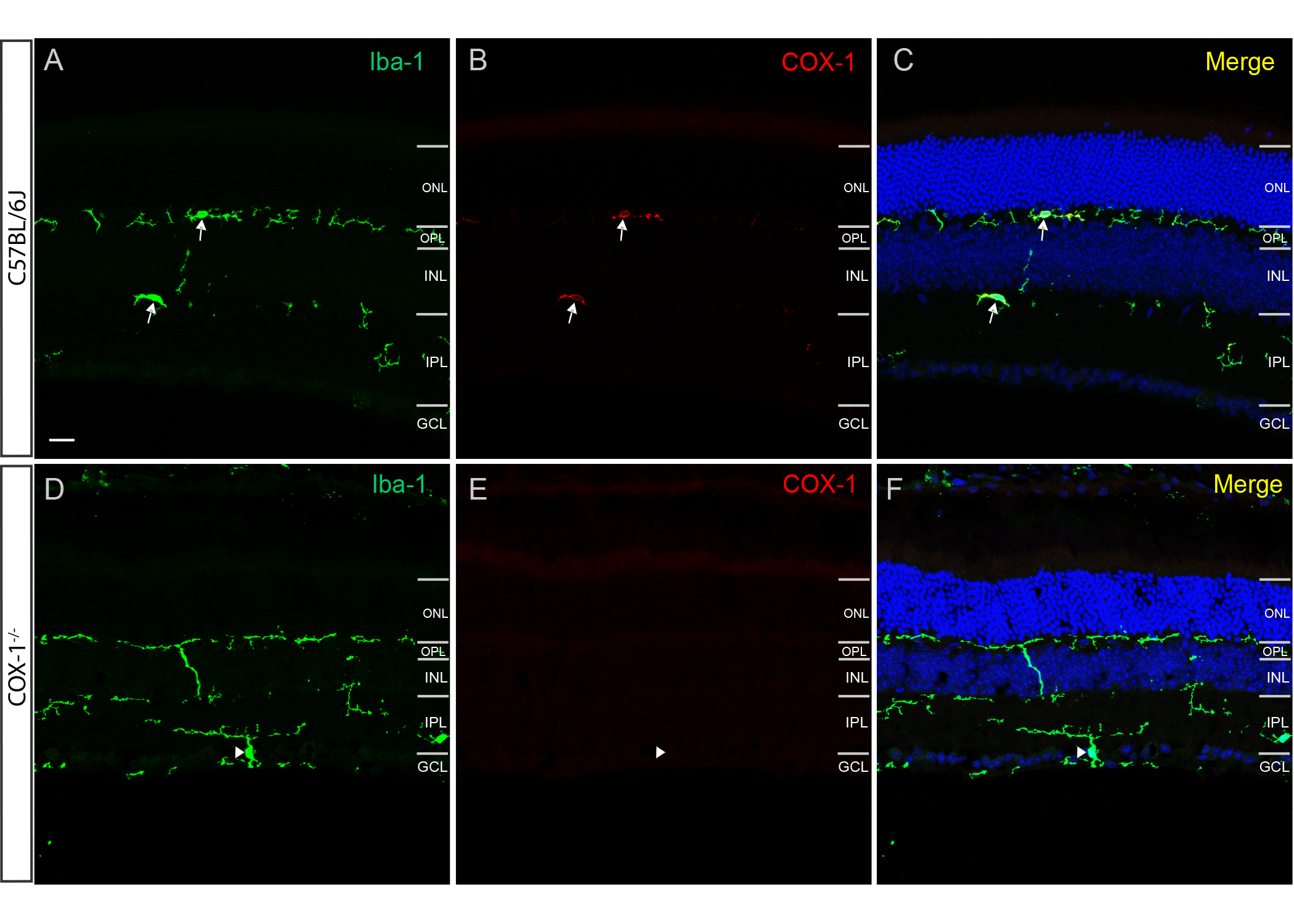

Supplement: Supplementary file 3 — Supplementary Figure 3. Retinal sections from the dorsal retina at about 1 mm away from the optic nerve head along the dorsal-ventral axis of adult C57BL/6J (A-C) and COX-1-/- (D-F) mice were stained with an anti-rabbit COX-1 antibody (red) and Iba-1 (green), a microglial and macrophage-specific marker. Cell nuclei were stained with 4′,6-diamidino-2-phenylindole (DAPI) (blue). Arrows indicate colocalization between microglia (green) and COX-1 signal (red) in the C57BL/6J mouse retina (A-C), while an arrowhead points to a microglia cell without any COX-1 signal in the COX-1-/- mouse retina (D-F). ONL, outer nuclear layer; OPL, outer plexiform layer; INL, inner nuclear layer; IPL, inner plexiform layer; GCL, ganglion cell layer. Scale bar: 20 μm. (TIF 2.83 MB) [file 12974_2020_1993_MOESM3_ESM.tif]
